# Sub-Frame Contact-Onset Estimation in a Self-Calibrated BJT Thermal Pixel Array Using a Four-Frame erfc Template

**DOI:** 10.3390/s26134074

**Published:** 2026-06-26

**Authors:** Yinglei Ma, Fei Xiao

**Affiliations:** College of Smart Materials and Future Energy, Fudan University, Shanghai 200433, China; feixiao@fudan.edu.cn

**Keywords:** BJT thermal pixel array, thermal-contact tactile sensor, contact-onset estimation, complementary error function, grid-initialized semi-analytic parameter estimation, Cramér–Rao bound, Bayesian inference, thermal sensor self-calibration

## Abstract

Low-cost bipolar-junction-transistor (BJT) thermal pixel arrays provide robust, force-free contact sensing for tactile skins, but their slow frame rate confines contact-timing resolution to the inter-frame interval—252 ms at the 4 Hz rate of the 16 × 16 array studied here—well below the needs of contact-aware control. We propose a four-frame complementary-error-function (erfc) template, derived from one-dimensional semi-infinite heat conduction, that jointly estimates the contact amplitude, the thermal-diffusion parameter, and the sub-frame contact-onset offset (*τ*_1_), solved by a grid-initialized semi-analytic Levenberg–Marquardt scheme (Path A) at deterministic single-pass cost. On 42 contacts from five subjects, the per-contact Cramér–Rao lower bound for *τ*_1_ is 16.2 ms, and the empirical cross-contact dispersion is 83.5 ms; both are internal, model-derived quantities, since no synchronised external timing reference was available. A two-layer rejection pipeline separates 19/19 valid contacts from 2/2 hardware faults; transfers to four held-out subjects (23/23) without retuning; attains an overall AUC of 0.878 on a five-class synthetic disturbance library—ramp and saturating-exponential remain acknowledged failure modes; and rejects 5/6 disturbance trials in a real-airflow stress session. Larger independent cohorts and externally synchronised timing validation remain parameters for future work.

## 1. Introduction

Reliable, inexpensive contact detection is a prerequisite for tactile skins in collaborative robots, assistive devices, and smart surfaces, where every square centimetre of sensing area must remain cheap, robust, and electrically quiet. Bipolar-junction-transistor (BJT) thermal pixel arrays meet these constraints by detecting contact through the conductive thermal contrast between warm skin and the cooler substrate: they need no mechanically active force-sensing element and are inexpensive when driven by simple readout application-specific integrated circuits (ASICs) [1,2]. Each pixel exploits the temperature-dependent base-emitter voltage (dV_BE/dT ≈ −2 mV/°C) to transduce local skin heating. The price of this simplicity is twofold: low-cost arrays suffer post-deployment thermal drift and inter-pixel non-uniformity [3] and require online recalibration, and—more fundamentally—they run at frame rates near 4 Hz, so the contact instant is known only to within the 252 ms inter-frame interval. This paper asks how much timing information can be recovered from such a slow sensor by exploiting the physics of heat conduction, and it shows that a four-frame physical template narrows the onset estimate to the sub-frame regime without any hardware change.

Positioning relative to other tactile pathways. Recent multimodal e-skin work emphasises piezoresistive, capacitive, triboelectric and biomimetic transducers integrated with vision [4,5,6,7]. The thermal-contact pathway has a narrower niche: intrinsic electromagnetic-interference (EMI) immunity, but low temporal bandwidth (a 4 Hz BJT array cannot resolve fast slip handled natively by piezoelectric/triboelectric sensors). The thermal-tactile literature focuses on material classification of touched surfaces [8,9], assuming contact is already segmented; this paper instead addresses the upstream contact-onset detection and sub-frame timing as a parametric estimation problem. High-frame-rate long-wave-infrared (LWIR) cameras (≥30 Hz) trivially solve sub-frame timing at order-of-magnitude higher per-pixel cost and optical-path requirements incompatible with a flat tactile-skin geometry; the BJT-array-plus-algorithm combination is a complementary low-cost design point, not a replacement.

Given a self-calibrated array (per-pixel baseline subtraction, Section 3.1; post-deployment peak-to-peak non-uniformity 0.322 °C → <0.1 °C), the design goals are as follows: (i) physically grounded in an analytic heat-conduction model; (ii) low function-evaluation count; (iii) able to flag non-erfc thermal disturbances via a per-contact shape-conformity gate (acknowledged failure modes are reported in Section 7.9); and (iv) tolerant to occasional sensor-node failures.

The semi-infinite-body solution of the heat equation gives surface-temperature rise of the form *A* · erfc(*B*/√*t*) [10,11], with *A* as the steady-state temperature contrast, and *B* as a thermal-diffusion parameter (units s^1⁄2^). For a four-frame observation window, we fit

(1) $$y(\tau_{k})=T_{env}+A\cdot erfc(\frac{B}{\sqrt{\tau_{1}+(k-1)\cdot \Delta t}})+n(\tau_{k}),\;\;k=1,\;\ldots ,\;4$$

where Δ*t* ≈ 252 ms, *τ*_1_ is the unknown sub-frame offset between actual contact onset and the first observation frame, and n is zero-mean Gaussian noise. Joint estimation of (*A*, *B*, and *τ*_1_) avoids manual onset annotation; *τ*_1_ implicitly recovers a sub-frame timestamp. Inferring fast-time-resolution events from slow-sampling sensors via parametric physical models is a recurring pattern (sub-sample ultrasonic time-of-flight, MIMO radar sub-Nyquist sampling, and physics-informed inverse heat-conduction [12]). A naive 4 Hz grid implies 252 ms timing; joint erfc estimation reports an internal CRLB *σ*_*τ*_1_,min ≈ 16.2 ms (an in-model Cramér–Rao lower bound under the empirical noise floor; realised empirical cross-contact dispersion, 83.5 ms; Section 6.3.4). Practical relevance: Contact-onset timing at the inter-frame scale is required when temporally fusing the thermal stream with a faster co-located capacitive/piezoelectric primary detector—sub-frame *τ*_1_ aligns (*A*, *B*) with the primary-detector onset within a single observation frame, while 252 ms frame-level timing cannot. We treat sub-frame timing as an enabling capability for downstream fusion rather than a standalone deployment claim (Section 7.5). A second contribution is a two-stage rejection pipeline addressing hardware-level node failures and physical-disturbance misclassification, outputting a three-class label (VALID_CONTACT/FAULT_CONTACT/DISTURBANCE) for fault-tolerant downstream control [13,14].

Contributions. Evaluated on a five-subject, six-session cohort (Table 1), this paper makes four contributions: C1, A four-frame erfc template that jointly recovers (*A*, *B*, and *τ*_1_) from a 1 s window, with a per-contact internal CRLB of *σ*_*τ*_1_,min ≈ 16.2 ms and an empirical cross-contact dispersion of 83.5 ms; C2, a grid-initialized semi-analytic solver (Path A) combining a deterministic 1800-point (*B*, *τ*_1_) grid with analytic elimination of *A* and a single LM refinement and that it is numerically identical to multi-start LM, and to a standard constrained solver (Section 6.12), at deterministic single-pass cost; C3, a CRLB/Fisher-information-matrix (FIM) analysis identifying *N* = 4 frames as the minimum well-conditioned operating point under the current noise floor and latency trade-off; and C4, an *R*^2^ physical-consistency gate whose overall AUC is 0.878 across all five synthetic disturbance classes (subset AUC 0.989 over the three physically representative classes; ramp and saturating-exponential are acknowledged failure modes, Table 2), complemented by 5/6 rejection on a 30 min real-airflow benchmark.

Section 2 reviews related work; Section 3 derives the model; Section 4 introduces the four estimators (Paths A–D) and the sensor-fault layer; Section 5 describes the experiment; Section 6 presents results; Section 7 discusses scope; Section 8 concludes.

## 2. Related Work

### 2.1. Thermal Contact and Tactile Sensing

Low-resolution thermal arrays for tactile sensing have been explored using thermopile arrays [15], pyroelectric pixel grids [15], and BJT thermal-contact pixel arrays [1,3]. The work presented here is conceptually close to BJT-based smart temperature sensor arrays [3,16], which use simple frame-mean thresholding or hardware redundancy without explicit physical models. Multimodal e-skin platforms reported in 2024–2026 [4] integrate thermal sensing with capacitive pressure or vision, but most have not formalised the contact-onset estimation problem at the sub-frame level. The static per-pixel non-uniformity of BJT-based low-resolution thermal arrays has been well-documented [3,16], and our per-pixel baseline subtraction addresses time-varying drift through a standard per-pixel self-calibration. Related self-calibration methods for CMOS temperature sensors include hybrid thermal-diffusivity/resistor designs [17] that use an on-chip reference instead of a spatial-region structure.

### 2.2. Sub-Sample/Sub-Frame Parameter Estimation in Slow Sensor Systems

The Sub-Sample Timing Estimation Problem in ultrasound and radar is relatively well-known. Sub-sample interpolation in the frequency domain can be used to achieve bias-free recovery of time-of-flight beyond the sampling grid in ultrasonic time-of-flight estimation [18], and auxiliary-function-based iterative updates extend this concept to estimate multiple delays simultaneously [19]. In radar parameter estimation, the same Cramér–Rao framework guides analytic sub-Nyquist samplers to decouple angle-of-arrival and delay-Doppler estimation, approach the information-theoretic bound [20], and Fisher-information analyses at low signal-to-noise ratios inform system-level design choices such as filter shape and sampling cadence [21]. The methodology applied to all of them is analytic parametric inference based on the Cramér–Rao bound; thus, it can also be used for our BJT thermal pixel array despite the differences in physical modalities, and this motivates the development of the four-frame analytic estimator in Section 4.

### 2.3. Physics Model-Based Estimation Versus Learned Classifiers

Analytic heat-conduction templates for contact analysis can be traced back to early thermography studies [11]. The closed-form erfc solution for one-dimensional heat conduction in a semi-infinite body is well-known [16]. Bai, Chen, Healy, Kemp, and Bhattacharjee [8] used a semi-infinite-body model to classify thermal-tactile materials at different initial sensor temperatures, but their method assumed annotated contact-onset and did not jointly estimate it with thermal parameters. Ma and Zhang [9] use a discrete transient model for contact-based thermosensation and also have externally provided contact-onset annotations. Recently, physics-informed neural networks (PINNs) have solved inverse heat-conduction problems with a small number of measurement points [12], and it has been shown that data-light, physics-grounded estimators are competitive with deep-learning alternatives for slow-thermal inverse problems where the physics is well-modelled. Methodologically, this paper is close to the research on inverse heat-conduction problems (IHCP) [22,23], which aims to find the thermal properties or boundary conditions based on a small amount of temperature data. The particular case considered here is joint estimation of an amplitude, a dimensional diffusion parameter, and a sub-frame time offset for *N* = 4 frames covering a single second; due to the analytic invertibility of the erfc template and the four-parameter match between *N* = 4 observations and (*A*, *B*, and *τ*_1_) plus a known *T*_env, this is a small-sample, well-posed instance of the general IHCP class, and it differs from the canonical ill-posed boundary-flux reconstruction problems addressed in [22,23]. Among them, the new research eliminates the contact-onset assumption that all the above methods rely on by jointly estimating (*A*, *B*, and *τ*_1_) from the same observation window with an analytic template-based estimator, thereby avoiding manual or external annotation steps and providing a within-model sub-frame timing parameter (interpreted in the frame-aligned convention introduced in Section 3.3).

### 2.4. Open-Set Rejection, Sensor Fault Tolerance, and Edge-Deployed Inference

Many sensors are used to carry out a residual-based physical consistency test and discard out-of-model disturbances [2]. The first type of statistical measure that is not based on residuals is *R*^2^. The literature on open-set recognition (OSR) and out-of-distribution (OOD) detection has grown into a mature subfield [5,13], and it has been established that OSR/OOD detection is now necessary for safety-critical perception systems, as some labelled anomalies may be available at training time, but the distribution of anomalies in the test phase can be broader. Surveys on sensor fault detection and diagnosis in robotic systems [14] have listed many types of failure modes for mechanically deployed sensor arrays and proposed adding fault detection to adaptive observers and predictive trajectory models. We use *R*^2^ as a one-class detection statistic for thermal tactile signals and compare it with amplitude- and slope-based baselines, and add a sensor-fault detection layer below it to provide a three-class output of VALID_CONTACT, FAULT_CONTACT, and DISTURBANCE for fault-tolerant downstream control.

## 3. Sensor Model and Problem Formulation

### 3.1. Sensor Description

The sensor is a 16 × 16 BJT thermal pixel array with a frame rate of 4 Hz (inter-frame interval of Δ*t* ≈ 0.252 s), a nominal temporal noise of 0.092 °C per pixel, and 5× factory-specification post-deployment peak-to-peak non-uniformity prior to calibration. After per-pixel baseline subtraction, the per-pixel residual noise, *σ* (after frame-mean subtraction), is approximately 0.025 °C (empirical, see Section 6.3.1), and inter-pixel non-uniformity has been reduced to within the factory’s ±0.1 °C specification.

The array used in this study is a custom in-house prototype developed by our group for self-calibrated contact-thermal sensing research, rather than a commercial product; the readout, calibration, and acquisition firmware were likewise developed in-house. Each pixel is a silicon bipolar-junction transistor whose base-emitter voltage decreases by approximately 2 mV per degree Celsius, and the readout application-specific integrated circuit (ASIC) converts this voltage change into a digital temperature value after on-chip biasing and gain. The sensor is a contact-thermal type; that is to say, changes in temperature occur due to the conduction of heat from an object in contact with the substrate, not by radiative absorption. When a finger is placed on the array surface, heat flows from the warm finger pad through the substrate to the BJT junction, increases the local junction temperature, and thus generates a V_BE shift that is detected by the readout chain. The substrate-and-package thermal mass and the contact-side thermal resistance together determine the rise-time of the BJT-junction temperature response, and this is the physical origin of the erfc model introduced in Section 3.2.

The per-pixel baseline subtraction described above is a precondition for the sub-frame estimation developed in the following sections, not an optional refinement; its quantitative role is discussed in Section 7.2.

### 3.2. Heat-Transfer Model

At time *t* = 0, when a finger touches a thermal pixel, according to the one-dimensional heat conduction problem in a semi-infinite body with a constant surface temperature of *T*_s = *T*_finger, the temperature at depth *z* = 0 of the pixel will change according to the following [16]:

(2) $$T(t)\;-\;T_{env}\;=\;A\;\cdot \;erfc(\frac{B}{\sqrt{t}})$$

where *A* = *T*_finger − *T*_env is the steady-state temperature contrast, and *B* = *z*_eff/(2√*α*) lumps the effective thermal contact resistance and pixel depth into a single parameter (*α* is the substrate thermal diffusivity); dimensional analysis gives [*B*] = [*z*_eff]/[√*α*] = m/(m·s^−1⁄2^) = s^1⁄2^, so *B* has units of s^1⁄2^, and only the ratio *B*/√*t* in the erfc argument is dimensionless. Under the calibrated noise floor, this template fits the rise of the contact response in our data with an *R*^2^ > 0.95 (Section 6).

Empirically, our 19 Subject-A contacts give $\bar{B}$ = 0.570 ± 0.075 s^1⁄2^ (Section 6.3). Under three candidate substrate materials, the inferred *z*_eff = 2$\bar{B}$√*α* covers a three-order-of-magnitude range: bulk silicon (*α* ≈ 9.0 × 10^−5^ m^2^/s) gives *z*_eff ≈ 10.8 mm, inconsistent with the 500 μm die thickness; epoxy encapsulation (*α* ≈ 1.2 × 10^−7^ m^2^/s) and silicone potting (*α* ≈ 1.0 × 10^−7^ m^2^/s) both give *z*_eff ≈ 0.36–0.39 mm, consistent with a sub-millimetre encapsulation layer over the die. We therefore present *B* as a phenomenological parameter capturing whatever effective thermal length scale dominates substrate-side transport, without committing to a specific material. Across this *α* range, the ratio √(*α*·*t*_obs)/*z*_eff stays approximately constant at ≈ 0.88 because both quantities scale with √*α*, so the semi-infinite assumption is itself material-independent in this regime; the four-frame window (*t*_obs ≤ 0.99 s) sits well inside the validity envelope, consistent with the observed *R*^2^ > 0.95 fit quality, and the model is expected to break down only at *t*_obs ≳ 5 s. Direct measurement of the encapsulation diffusivity would convert *B* into an absolute length scale; this is deferred, and neither the contact-onset estimation nor the two-layer rejection analysis depends on it, as both are derived from the analytical erfc shape without requiring an absolute length scale for *B*.

### 3.3. Four-Frame Observation Window with Sub-Frame Onset

Let *t*_c be the actual contact-onset time, and generally it falls between two consecutive sample times. Let *τ*_1_ = *t*_1 − *t*_*c* ∈ [0, Δ*t*] be the offset from contact-onset to the first observation frame. Then the four-frame observation, y_k for *k* = 1, …, 4, is given by Equation (1). We present two coordinate systems in this paper: the model *τ*_1_ (defined here) is a non-negative offset from contact onset to the first observation frame; for simplicity in reporting in Section 6, we use a frame-aligned convention where *t* = 0 is set at the first detected frame, and thus we report *τ*_1_, frame = −*τ*_1_, so negative reported values indicate that the contact onset occurred before the first detected frame by an amount less than the inter-frame interval, Δ*t*. Both conventions describe the same physical event and yield identical estimator behaviour; therefore, we use the model convention *τ*_1_ ≥ 0 in the derivation and the frame-aligned convention in the results. Direct least-squares fitting of (1) over (*A*, *B*, and *τ*_1_) yields a joint estimator that simultaneously infers contact-onset (via *τ*_1_) and the thermal parameters (*A* and *B*). Based on the CRLB analysis in Section 4.3, we select *N* = 4 frames; it is the smallest such *N* that does not lead to an order-of-magnitude increase in any single parameter.

## 4. Method

### 4.1. Path A: Grid-Initialized Levenberg–Marquardt Solver with Analytic Amplitude Reduction

Direct nonlinear least-squares optimization over (*A*, *B*, and *τ*_1_) with multi-start LM is expensive and may converge to a local minimum. Path A exploits the analytic-elimination structure of Equation (1):

Step 1 (Grid). Discretize (*B*, *τ*_1_) with *B* ∈ {0.05, …, 1.20} (60 points) and *τ*_1_ ∈ {0.05 Δ*t*, …, 0.95 Δ*t*} (30 points), giving 1800 grid points.

Step 2 (analytic-*A* reduction). At each grid point, *A* is eliminated by the linear least-squares normal equation $\hat{A}$(*B*, *τ*_1_) = ⟨y − *T*_env, e⟩/⟨e, e⟩, with e_k = erfc(*B*/√(*τ*_1_ + (*k* − 1) Δ*t*)); evaluate *R*^2^ = 1 − SSR/SST and select the maximum.

Step 3 (LM refinement). One LM run from the grid optimum, typically converging in 6 iterations [24,25].

Total cost per contact is 1800 lightweight grid evaluations + 6 heavyweight LM iterations, vs. 109 heavyweight LM iterations for multi-start LM. On the 19 Subject-A contacts, the two methods agree to max |Δ*A*| = 4.4 × 10^−5^ °C and max |Δ*B*| = 1.1 × 10^−6^. The term “closed-form” applies strictly to the analytic-*A* reduction at each grid point; the overall procedure is hybrid grid + LM, not a globally closed-form estimator. This is a platform-independent computational-work measure; wall-clock benchmarking on x86/ARM Cortex-M/RISC-V is a subject for future work.

### 4.2. Path B: Generalised-Likelihood-Ratio-Test (GLRT) Detection

Given the four-frame observation, y, the generalized likelihood ratio test (GLRT) statistic for testing H_0_ (no contact) vs. H_1_ (contact present with parameters (*A*, *B*, and *τ*_1_)) is as follows:

(3) $$2\Lambda \;=\;\frac{SST\;-\;SSR(\hat{A},\;\hat{B},\;{\hat{\tau }}_{1})}{\sigma^{2}}$$

Under H_0_ and Wilks’ theorem, 2*Λ* is asymptotically *χ*^2^(2) distributed, and thus a parametric threshold of *τ*_*α* = *χ*^2^_2(1−*α*) can be obtained for any false-alarm rate, *α*. Due to a small sample size (*N* = 4) and a constrained parameter space, it is not feasible to achieve asymptotic accuracy; thus, we have empirically tested the detection performance (Section 6.2). The *R*^2^ value of Section 4.4 is employed as a secondary check for physical consistency that is less sensitive to non-erfc disturbances, and the relationship between *R*^2^ and 2*Λ* is discussed in Section 7.

### 4.3. Path C: CRLB Analysis

The Fisher Information Matrix for the joint (*A*, *B*, and *τ*_1_) estimation under *N* observations and per-sample noise, *σ*, is derived by direct differentiation of Equation (1). The diagonal CRLB elements *σ*^2^_*A*,min, *σ*^2^_*B*,min, and *σ*^2^_*τ*_1_,min are functions of *N*, *A*, *B*, and *τ*_1_; they are calculated using the empirical mean parameters (Section 6.3). We will use bootstrapping and a *χ*^2^ test to determine whether the dispersion of the empirical sample across the 19 Subject-A contacts is consistent with the per-contact CRLB (Section 6.3.2).

### 4.4. Path D: Bayesian Laplace Approximation

Independent weakly informative priors: *A* ∼ N(6, 2^2^) °C; *B* ∼ TN(0.5, 0.2^2^) s^1⁄2^ positive-truncated; *τ*_1_ ∼ U [0, Δ*t*]. Prior widths are ≈ 4–5× the likelihood-determined posterior SDs (*σ*_*A*,post ≈ 0.451 °C, *σ*_*B*,post ≈ 0.040 s^1⁄2^), placing the analysis in the data-dominated regime (prior ≈ 4–5% of posterior precision; Section 6.5). Laplace approximation at the maximum a posteriori (MAP) estimate gives a Gaussian posterior; marginalised mass over H_1_ (*A* > 0, *B* > 0) gives P(contact|y).

### 4.5. Sensor-Fault Rejection Layer (Layer 1) and Two-Layer Pipeline

Mechanically deployed BJT arrays can saturate pixels under PCB flex from excessive contact pressure (Section 6.6). We add a pre-Layer-2 saturation gate on the same four-frame window:

(1) Layer 1 (Saturation count): Count pixels at the analogue-to-digital-converter (ADC) ceiling (≥60 °C; 65,535 LSB) across the four-frame window. If *N*_sat ≥ *N*_sat,thr (default 5), output FAULT_CONTACT (confirmed contact with unreliable parametric estimates).

(2) Layer 2 (Path A erfc fit): Joint estimation of (*A*, *B*, and *τ*_1_); if *R*^2^ < *R*^2^_thr (default 0.95) or *B* outside [*B*_min, *B*_max] = [0.10, 2.00], output DISTURBANCE. The aggregated cross-contact *F*-test of Section Aggregated Cross-Contact F-Test for the erfc Model (Subject A: *F* = 134.46, *p* = 2.2 × 10^−16^, ΔAIC = −335; held-out B–E: *F* = 170.46, *p* = 1.3 × 10^−20^, ΔAIC = −427) establishes that the sub-frame timing offset, *τ*_1_, is statistically supported within the erfc parameterisation. *R*^2^_thr = 0.95 was fixed a priori on the Subject-A *R*^2^ distribution (all *R*^2^ ≥ 0.99). The Section 6.6 Subject-A figures are therefore re-substitution; the held-out Subject-B–E cohort (23 contacts) provides one round of independent threshold-portability evidence (all 23 pass without retuning). See Section 7.9 (vii) for the formal limitation.

If both layers pass, output VALID_CONTACT. The three output classes—VALID_CONTACT, FAULT_CONTACT, and DISTURBANCE—together cover the failure-mode space.

### 4.6. Multi-Source Spatial Cardinality via Bayesian Model Selection

For multi-finger contacts, the array image at the steady-state portion of the contact event is modelled as a superposition of *K* Gaussian sources at unknown locations (*i*_l, *j*_l) and amplitudes, *A*_l, l = 1…*K*, plus an isotropic noise, *σ*_s:

(4) $$f(i,\;j;\;\theta_{K})\;=\;T_{env}\;+\;\sum_{l=1}^{K}A_{l}\;\cdot \;g(i\;-\;i_{l},\;j\;-\;j_{l})$$

where *g*(·) is the empirical single-finger response template extracted from isolated bright blobs of the 19 valid Subject-A contacts (a 9 × 9 patch with peak ≈ 5.8 °C and FWHM ≈ 5 pixels). Equation (4) approximates the convolution between *K* finger thermal contacts and the BJT pixel point-spread function. Cardinality *K* is selected by the Bayesian Information Criterion:

(5) $$K^{*}\;=\;{argmin}_{K}\;[N\;\cdot \;ln({\hat{\sigma }}^{2}(K))\;+\;(3K\;+\;1)\;\cdot \;ln(N)]$$

with *N* = 256 pixels, $\hat{\sigma }$^2^(*K*) = SSR(*K*)/(*N* − 3*K* − 1) from Nelder–Mead with multiple random restarts. To prevent artificial increases in *K* from model-misspecification residuals (real finger imprints leave ≈ 0.2 °C structural residual even after a correct-*K* fit), an extra *K* · ln(*N*) penalty is added: BIC′ = BIC + *K* · ln(*N*). This is a BIC-doubled-penalty heuristic (penalty coefficient 2 ln *N* per source rather than 2 ln(ln *N*) per source of the proper Hannan–Quinn criterion [26]); we use this stronger penalty empirically to suppress over-splitting and do not interpret it as a principled information-theoretic criterion. BIC′ keeps $\hat{K}$ = 1 for single-finger contacts; posterior P(*K*|y) follows from exp(−BIC′/2). $\hat{K}$ accuracy on synthetic single-finger trials is stable for penalty scaling, *c* ∈ [0.5, 4.0], at *σ*_eff = 0.092 °C (100% at *c* = 1.0 operating point; 71% at *c* = 0.25 due to over-splitting). A 2-D sensitivity sweep over (*c*, *σ*_eff) for multi-finger/higher-noise conditions is a subject for future work; the Section 6.10 result *K*_max = 3 should be read as conditional on *c* = 1.

## 5. Experimental Protocol

### 5.1. Hardware and Recording

**Figure 1:**
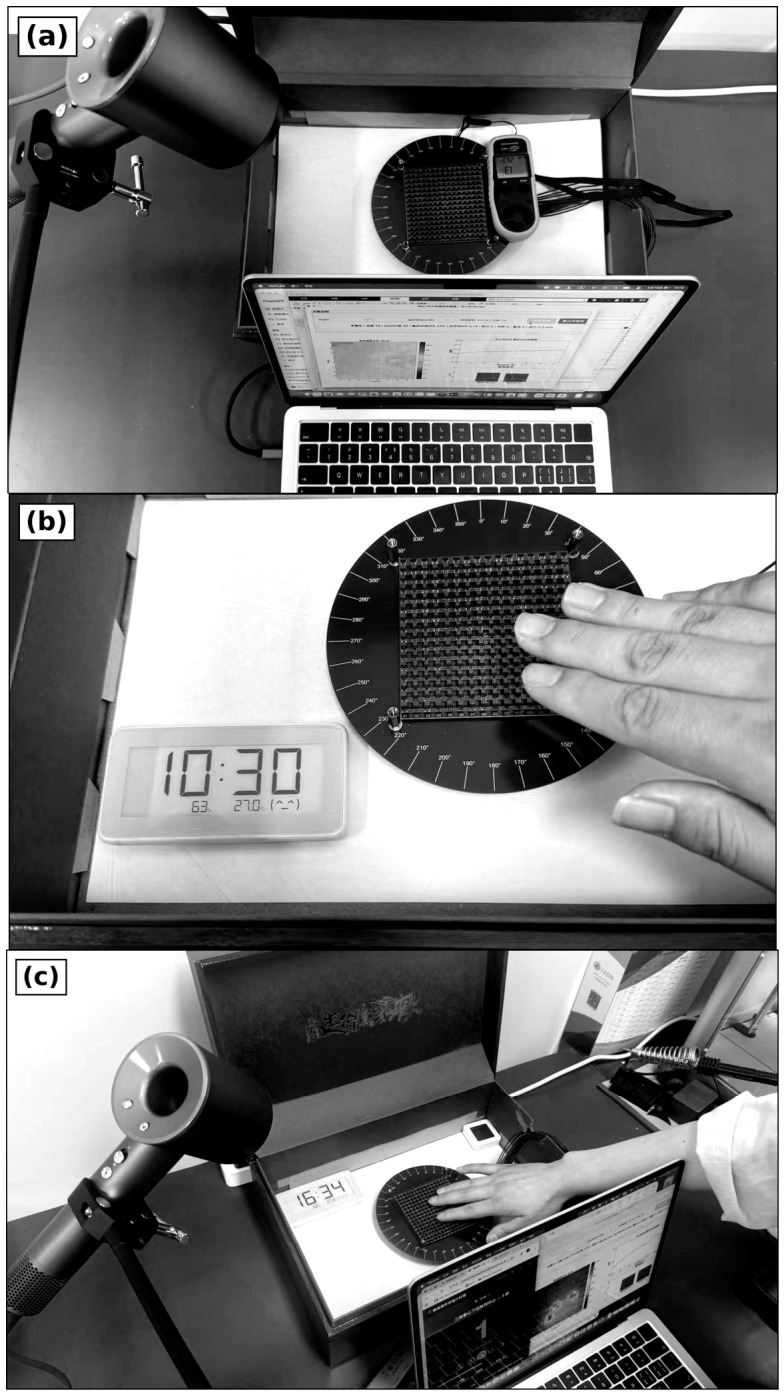
(**a**). Experimental setup: 16 × 16 BJT thermal pixel array on host PCB in a test bench at 24.8–26.0 °C, contact surface facing upwards, and read at 4 Hz over serial. (**b**). Hand-contact protocol. Subject performs a three-finger, palm-down press at the array centre for ≈ 5 s and then rests ≥ 90 s. Contact onset falls at a random sub-frame offset, *τ*_1_, from the 4 Hz sampling clock. (**c**). Real-airflow disturbance setup. Consumer hair dryer perpendicular to the array centre; surface airflow of 4.7 m/s (cool)/1.8 m/s (hot). Six trials in a 30 min session (3 cold + 3 hot, 5 min recovery between trials). Hot-air stream ≈ 40 °C at the array surface (measured by the anemometer’s integrated temperature sensor).

A 16 × 16 BJT thermal pixel array operating at room temperature was selected (Figure 1), and it had a nominal frame rate of 4 Hz and an inter-frame interval of 252 ms. The ambient temperature during the observation period was 27.3 °C. A per-pixel baseline subtraction was performed using the first ~60 s of each session (subtracting the temporal mean over the baseline window from every pixel before analysis), and the same physical array was used for all subjects and sessions. All parameter estimation, model fitting, and statistical analyses were implemented in Python 3 (Python Software Foundation, Wilmington, DE, USA) using the NumPy and SciPy scientific-computing libraries.

### 5.2. Multi-Subject Contact Protocol

Five subjects, six sessions, and unified pressing protocol: Three-finger (index + middle + ring) presses at the array centre, 5 s contact, ≥90 s inter-contact interval. *T*_hand and *T*_env were recorded once per session with a 1 K-class thermistor at the index fingertip and 30 cm from the array, after a 30 s wait. Recorded *T*_hand is a session-level value; per-contact drift is absorbed in the mixed-effects residual variance (Section 6.7).

Subject A contributed the 47.4 min primary recording (21 attempted, 19 valid; Section 6.6) and served as the algorithm-development set for fixing the four hyperparameters—Path A (*B*, *τ*_1_) grid, *N*_sat,thr = 5, *R*^2^_thr = 0.95, and *σ*_noise = 0.025 °C—before any analysis of Subjects B–E. The Subject-B–E cohort is a held-out portability check, not an independent population validation.

### 5.3. Open-Set Disturbance Library: Synthetic

To evaluate the robustness of the detector to non-erfc disturbances, 1250 four-frame samples in five categories (250 per category) with amplitudes of Δ*T* = 1–5 °C designed to overlap the contact Δ*T* range were synthesized:

1. Step: A sudden increase in temperature within the four-frame window.
2. Ramp: A linear increase in temperature over the four frames.
3. Square: Square-wave oscillation of the baseline and elevated levels.
4. Oscillation: Sinusoidal modulation, frequency 0.5–2 Hz.
5. Saturated exponential: Shape of 1 − e^−kt^, for the start of convective heating.

All synthetic disturbances are added to the real baseline traces from the calibrated array (*σ*_noise = 0.025 °C), and the realistic noise floor of the array is preserved. Only these samples will be used for the detector benchmark, and they will not be used in parameter-estimation evaluation.

### 5.4. Real-Airflow Disturbance Recording

To supplement the synthetic library with a physical benchmark, we recorded a separate 30 min Subject-A session of six forced-convection trials (3 cold-air + 3 hot-air) using a consumer hair dryer perpendicular to the array centre. Airflow velocity at the array surface was 4.7 m/s (cool) vs. 1.8 m/s (hot—the heating coil introduces flow impedance). The hot-air stream temperature, measured near the array surface by the temperature sensor integrated in the anemometer (the same instrument used for the airflow-velocity measurements; not independently calibrated), was approximately 40 °C; because this value was not closed-loop controlled, the hot-air trials serve as a qualitative real-disturbance source rather than a controlled thermal set-point. Cold air at 4.7 m/s remains close to ambient (small Δ*T*), while hot air at 1.8 m/s carries a source temperature far above ambient (larger Δ*T*). Recording sample rate was 3.84 Hz (slightly below the 4 Hz nominal Subject-A rate due to acquisition-pipeline jitter).

Trials were nominally scheduled every 5 min, allowing full thermal recovery after each disturbance. The nominal duration of the blow was 15 s per trial; operator-driven on/off timing introduced a timing jitter of ±2–25 s relative to the scheduled instant, which was not used in the subsequent analysis because the rejection pipeline operates only on the per-frame signal. The ambient environment temperature was 25.2 °C at all times, and the array baseline recorded this.

### 5.5. Automatic Rejection Results (Motivating Section 6.6)

The 47.4-min Subject-A recording contained 21 protocol-attempted contacts; this recording was terminated after contact #21 by a separate user-induced over-pressure event (frames, 11,263 onwards; 92 saturated trailing frames) that was excluded by the same conservative criterion as in Section 3.1 and is not counted as a contact attempt because no new contact was initiated. Frame-mean thresholding was performed on the retained 11,261 frames, and 21 candidate contact events matching the protocol attempts were detected. Among them, two events (at frames 1743 and 9021, corresponding to recording times of 7.28 min and 37.69 min) coincided with mechano-electrical PCB-flex saturation in pixel columns 11 and 13, and there were 122–128 saturated pixel-frames within the four-frame observation window. The remaining 19 events had zero saturated pixels. The sensor-fault rejection rule in Section 4.5 (Layer 1) automatically classifies these two populations: events with *N*_sat ≥ *N*_sat,thr are considered FAULT_CONTACT, and others are forwarded to Layer 2. All the following parameter analyses use the 19 VALID_CONTACT events. Layer-1 statistics are shown in Section 6.6.

## 6. Results

### 6.1. erfc Model Fit and Path A Speed

**Figure 2:**
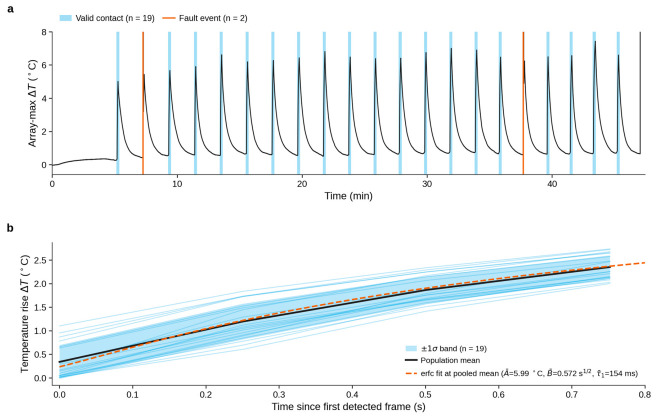
(**a**) Subject-A recording, 47.4 min: 19 valid contacts (light bands) and 2 sensor-fault rejected events (red). (**b**) Nineteen contact rise curves at the peak pixel; black curve = population mean (*n* = 19), grey band = ± 1*σ*, dashed curve = erfc fit at population-mean ($\bar{A}$ = 5.98 °C, $\bar{B}$ = 0.570 s^1⁄2^). Mean per-contact *R*^2^ = 0.999.

Figure 2 shows the full 47-min Subject-A recording (Figure 2a); the 19 retained contacts are shown as light-shaded vertical bands, and the two rejected contacts (#2 and #17 in the original numbering) are hatched in red—these were rejected by Layer 1 and are presented here for visualization. Figure 2b shows the per-pixel temperature rise curves for the 19 contacts at their most-responsive pixels, as well as the population mean (black) and the erfc fit based on the population-mean parameters (dashed curve). The average fit *R*^2^ for all 19 Subject-A contacts is 0.999.

#### Aggregated Cross-Contact F-Test for the erfc Model

We supplement the per-contact *R*^2^ with an aggregated *F*-test pooling residuals across all 19 Subject-A contacts (76 total observations, 19 effective residual DOF after profiling). The nested test of the *τ*_1_-free erfc model against its *τ*_1_ = 0 restriction gives *F*(19, 19) = 134.46 (*p* = 2.2 × 10^−16^) and ΔAIC = −335, where the numerator df = 19 counts one extra *τ*_1_ per contact, and the denominator, df = 19 = 76 − 57, is the unrestricted-model residual DOF (76 observations, 3 × 19 = 57 free parameters), establishing that the sub-frame timing offset, *τ*_1_, is statistically supported. On the held-out Subject-B–E cohort (23 contacts, 92 observations), the same test gives *F*(23, 23) = 170.46 (*p* = 1.3 × 10^−20^), with ΔAIC = −427, confirming this transfers to subjects not used in algorithm development.

**Figure 3:**
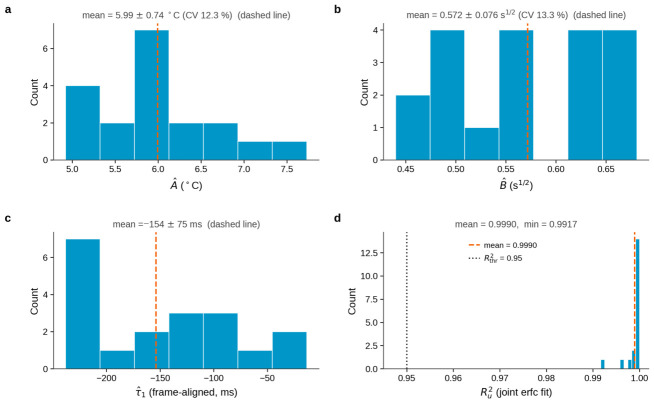
Distribution of joint-fit parameters across the 19 Subject-A contacts. (**a**) Estimated $\hat{A}$ histogram (5.98 ± 0.74 °C, CV 12.4%). (**b**) Estimated $\hat{B}$ histogram (0.570 ± 0.075 s^1⁄2^, CV 13.2%). (**c**) Estimated $\hat{\tau }$_1_ in the frame-aligned convention (−154 ± 83.5 ms; negative values indicate contact onset preceded the first detected frame). (**d**) Per-contact *R*^2^ histogram of the joint erfc fit (mean 0.9990, min 0.9917; *R*^2^_thr = 0.95 marked).

Path A is functionally identical to multi-start LM but uses a deterministic 1800-point grid + analytic-*A* reduction + six-step LM refinement (Section 4.1). The two methods agree to max |Δ$\hat{A}$| = 4.4 × 10^−5^ °C and max |Δ$\hat{B}$| = 1.1 × 10^−6^ s^1⁄2^ over all 19 Subject-A contacts; the function-evaluation classes differ by an order of magnitude (1800 grid + six heavy LM vs. 109 heavy LM iterations). The per-contact distributions of the three estimated parameters across the 19 Subject-A contacts are shown in Figure 3.

### 6.2. Open-Set Disturbance Benchmark

**Figure 4:**
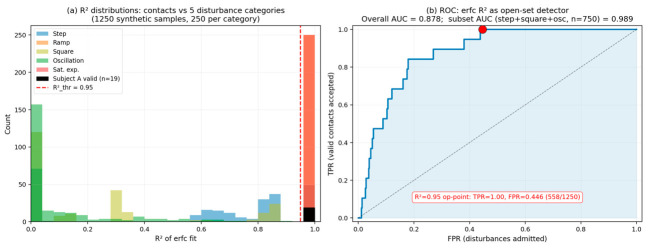
Open-set disturbance benchmark on the 1250-sample synthetic library. (**a**) *R*^2^ distributions: 19 Subject-A valid contacts (black) vs. 5 disturbance categories—step (blue), ramp (orange), square (yellow), oscillation (green), and saturating exponential (red) (each *n* = 250)—with the *R*^2^_thr = 0.95 decision line (red dashed). (**b**) Receiver operating-characteristic (ROC) curve of the per-contact *R*^2^ used as a coarse disturbance gate (not a deployment-grade open-set classifier): overall AUC = 0.878 (5 classes pooled, *n* = 1250); subset AUC = 0.989 over the physically representative classes (step + square + oscillation, *n* = 750). The *R*^2^ = 0.95 operating point (red marker) gives TPR = 1.00 (all 19 contacts pass) and FPR = 0.446 (558/1250 disturbances admitted, dominated by ramp and saturating-exponential, which are an acknowledged failure mode of the 4-frame *R*^2^ statistic; see Section 7.9 (ix)).

We evaluated the Path A *R*^2^ statistic on the 1250-sample synthetic disturbance library (Section 5.3, Figure 4) using the 19 Subject-A valid contacts as the H_1_ distribution. Per-class AUC: step = 0.97, square = 1.00, oscillation = 1.00, ramp = 0.85, and saturating exponential = 0.57. The first three classes (*n* = 750) yield a subset AUC = 0.989; the latter two (*n* = 500)—particularly saturating-exponential at AUC ≈ 0.57—are an acknowledged failure mode of the four-frame *R*^2^ statistic. The four-frame synthetic realisation of ramp and saturating-exponential disturbances does not span the natural ≥ 40-frame temporal extent of the physical phenomena they were intended to proxy; over four frames a slow ramp and a slow erfc rise are mutually indistinguishable in shape. We treat the subset AUC = 0.989 as a physically representative subset metric, not a deployment-level open-set robustness claim.

### 6.3. Per-Contact Precision: CRLB Analysis

#### 6.3.1. CRLB at the Subject-A Primary Operating Point

At Subject-A primary operating point ($\bar{A}$ = 5.98 °C, $\bar{B}$ = 0.570 s^1⁄2^, $\hat{\tau }$_1_ = 154 ms in the model coordinate, *N* = four frames, Δ*t* = 252 ms, *σ*_noise = 0.025 °C from the Path A fit residuals) the per-contact CRLB values are *σ*_*A*,min = 0.324 °C, *σ*_*B*,min = 0.028 s^1⁄2^, *σ*_*τ*_1_,min = 16.2 ms. The 16.2 ms timing CRLB is 15.5× smaller than the 252 ms sampling step—this is an internal Cramér–Rao lower bound under the empirical noise model, not an externally calibrated timing accuracy (Section 6.3.4).

#### 6.3.2. Empirical-to-CRLB Ratios (Bootstrap CIs)

**Figure 5:**
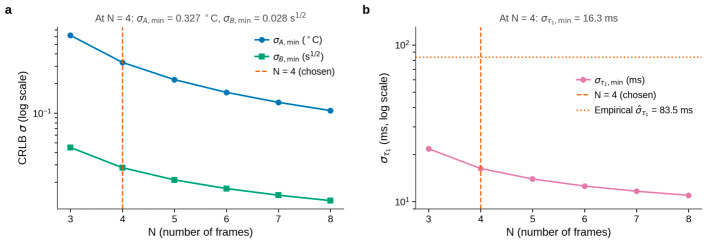
Cramér–Rao bound vs. frame count *N* at the Subject-A mean operating point ($\bar{A}$ = 5.99 °C, $\bar{B}$ = 0.572 s^1⁄2^, $\bar{\tau }$_1_ = 154 ms). (**a**) *σ*_*A*,min (°C, blue) and *σ*_*B*,min (s^1⁄2^, green) vs. *N* on a logarithmic scale; the order-of-magnitude drop from *N* = 3 to *N* = 4 identifies *N* = 4 as the smallest well-conditioned operating point (red dashed line). (**b**) *σ*_*τ*_1_,min (ms) vs. *N* on a logarithmic scale, with the empirical cross-contact dispersion $\hat{\sigma }$_*τ*_1_ ≈ 83.5 ms (bootstrap mean of Section 6.3.2) shown as a horizontal reference line for comparison.

Figure 5b reports the empirical sample standard deviation of ($\hat{A}$, $\hat{B}$, $\hat{\tau }$_1_) across the 19 Subject-A contacts, with Bootstrap 95% confidence intervals: $\hat{\sigma }$_*A* = 0.74 °C [0.56, 0.94], $\hat{\sigma }$_*B* = 0.075 s^1⁄2^ [0.057, 0.097], $\hat{\sigma }$_*τ*_1_ = 83.5 ms [56, 116]. The ratios to the per-contact CRLB are 2.28×, 2.68×, 5.2× respectively. *χ*^2^ consistency tests of $\hat{\sigma }$ = *σ*_min strongly reject H_0_ for all three (*χ*^2^ ≫ critical at df = 18; *p* < 10^−12^); the cross-contact dispersion is dominated by between-contact variability, not within-contact noise—see Section 6.3.4 for the constrained-estimator characterisation.

#### 6.3.3. FIM Eigenvalue and Parameter-Coupling Analysis

**Figure 6:**
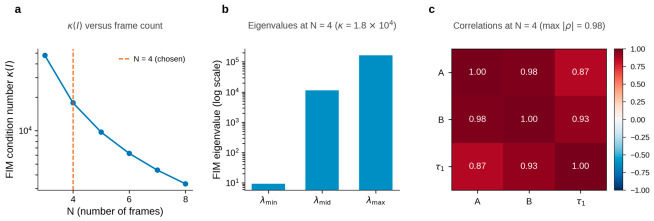
Fisher Information Matrix (FIM) analysis at the Subject-A mean operating point ($\bar{A}$ = 5.99 °C, $\bar{B}$ = 0.572 s^1⁄2^, $\bar{\tau }$_1_ = 154 ms, *σ*_noise = 0.025 °C). (**a**) FIM condition number *κ*(I) vs. frame count, *N* (log scale); *N* = 4 yields *κ* ≈ 1.8 × 10^4^ (chosen operating point, red dashed line). (**b**) FIM eigenvalues *λ*_min, *λ*_mid, and *λ*_max at *N* = 4 on a log scale. (**c**) Joint parameter-correlation matrix at *N* = 4: *ρ*_*AB* = 0.98, *ρ*_*Aτ*_1_ = 0.87, and *ρ*_*Bτ*_1_ = 0.93 (max |*ρ*| = 0.98), confirming all three parameters are highly coupled at the operating point.

The FIM condition number is *κ* = ∞ at *N* = 2 (degenerate), 4.8 × 10^4^ at *N* = 3, 1.8 × 10^4^ at *N* = 4, 9.7 × 10^3^ at *N* = 5 (Figure 6a). The order-of-magnitude drop *N* = 3 → 4 identifies *N* = 4 as the smallest well-conditioned operating point. The parameter-correlation matrix at *N* = 4 (*ρ*_*AB* ≈ +0.98, *ρ*_*Aτ* ≈ +0.87, *ρ*_*Bτ* ≈ +0.93) shows all three highly coupled; “CRLB” denotes the joint Cramér–Rao bound (trace of I^−1^), and per-parameter *σ*_·,min characterize marginal precision under joint estimation. With *A* and *B* profiled out, *σ*_*τ*_1_,min increases from 16.2 to ~38 ms: ~60% of the *τ*_1_ precision depends on joint estimation of *A* and *B*.

Note on noise floor: CRLB values use *σ*_noise = 0.025 °C from the Path A fit residuals (rather than the 0.092 °C nominal-noise calibration baseline of Section 3.1); the fit-residual choice is more conservative as it captures all unmodelled variations beyond pure sensor noise. FIM condition numbers were recomputed with the exact Section 6.3.1 parameter values using double-precision numerical differentiation; values supersede earlier approximate estimates.

Scope of the *N* = 4 recommendation. The CRLB/FIM analysis treats the residual as zero-mean Gaussian within-model noise, while the empirical cross-contact dispersion is protocol-dominated (Section 6.3.4: 5.2× CRLB excess from finger approach/pressure/skin–substrate coupling/constrained-estimator boundary effects, not measurement noise). Increasing *N* would not reduce the protocol-driven component; the CRLB justification of *N* = 4 is a statement about parameter identifiability under the empirical noise floor, not about deployment timing accuracy. Protocol standardisation (controlled-pressure fixturing) or an external high-speed timing reference is required to reduce deployment scatter (Section 7.9 (viii)).

#### 6.3.4. Per-Contact Estimation Precision Versus Population Variability

The two quantities reported in this section are different and should not be conflated. First, the per-contact CRLB *σ*_*τ*_1_,min = 16.2 ms is the internal Cramér–Rao lower bound of the erfc-template fit under the empirical noise model—it is not an externally calibrated timing accuracy, because the current dataset lacks an independent high-speed timing reference (e.g., a 1 kHz thermistor or a high-speed thermal camera co-registered with contact onset). Second, the cross-contact sample standard deviation of ${\hat{\tau }}_{1}$ across the 19 Subject-A contacts is $\hat{\sigma }$_*τ*_1_ = 83.5 ms (CV 54%), which reflects protocol-level variability and constrained-estimator effects across repeated contacts.

The empirical $\hat{\sigma }$_*τ*_1_ is ≈ 5.2× the per-contact CRLB. We attribute this to (i) between-contact variability in finger approach/pressure/skin–substrate coupling, and (ii) constrained-estimator effects near the [0, Δ*t*) boundary. Monte Carlo characterisation at six *τ*_1_ operating points (1000 four-frame observations, each at $\bar{A}$ = 5.98 °C, $\bar{B}$ = 0.570 s^1⁄2^, and *σ*_noise = 0.025 °C) shows $\hat{\sigma }$_*τ*_1_ ≈ 1.0× CRLB in the bulk (*τ*_1_ = 154 ms cohort mean) and 2.27× CRLB at the lower boundary *τ*_1_ = 10 ms (+17.4 ms inward bias, 41% pinning). The Subject-A cohort has 4/19 contacts within 25 ms of a boundary, so the 5.2× excess reflects both protocol variability and the constrained-estimator floor. The 5.2× ratio is a within-model consistency result, not an external accuracy claim; a high-speed timing reference (1 kHz thermistor or high-frame-rate IR) is required before any deployment-level claim.

### 6.4. Algorithm Comparison: Four Estimators on the Same 19-Contact Dataset

**Figure 7:**
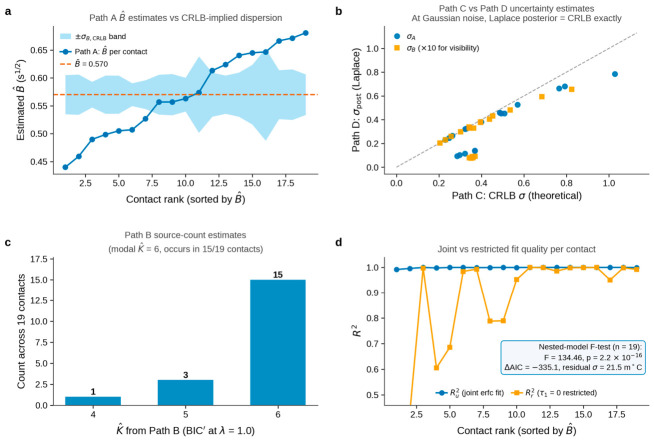
Four-algorithm parameter estimation comparison on the 19 valid Subject-A contacts. (**a**) Path A and $\hat{B}$ estimates per contact, sorted, with the ±*σ*_*B*,CRLB band overlaid; pooled $\bar{B}$ = 0.570 s^1⁄2^ (red dashed). The per-contact spread sits largely within the CRLB-implied band, indicating that between-contact variability dominates within-contact noise. (**b**) Per-contact comparison of the Path-C CRLB *σ* (evaluated at each fitted MLE) against the Path-D numerical Laplace posterior, *σ*_post, for *σ*_*A* (blue) and *σ*_*B* (orange, ×10 for visibility); the two agree closely for well-conditioned contacts and deviate only for the few contacts whose $\hat{\tau }$_1_ approaches the frame boundary. (**c**) BIC′ source-count estimate, $\hat{K}$, across the 19 contacts (BIC′ at *λ* = 1.0); the modal $\hat{K}$ = 6 occurs in 15/19 contacts. (**d**) Per-contact joint-fit *R*^2^_u vs. the restricted (*τ*_1_ = 0) *R*^2^_r, sorted by $\hat{B}$; the consistent gap motivates the aggregated nested-model *F*-test (*n* = 19: *F* = 134.46, *p* = 2.2 × 10^−16^, ΔAIC = −335.1, residual *σ* = 21.5 m°C).

**Figure 8:**
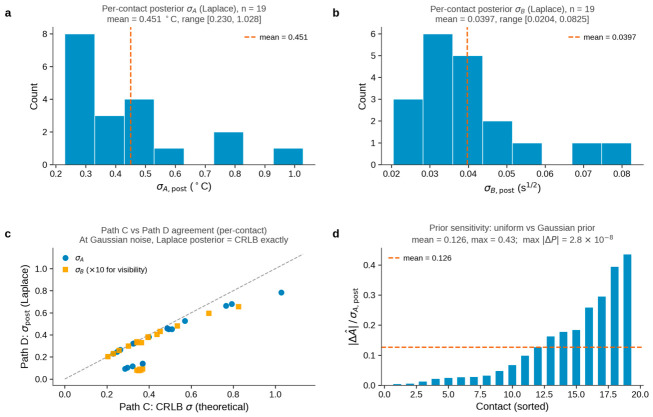
Path-D Bayesian uncertainty quantification on the 19 Subject-A contacts; here *σ*_post denotes the per-contact CRLB evaluated at each fitted MLE (Gauss–Newton Hessian), which the numerical Laplace posterior matches for well-conditioned contacts. (**a**) Distribution of per-contact *σ*_*A*,post: mean 0.451 °C, range [0.230, 1.028]. (**b**) Distribution of per-contact *σ*_*B*,post: mean 0.0397 s^1⁄2^, range [0.0204, 0.0825]. (**c**) Per-contact CRLB *σ* (Path C) vs. numerical Laplace posterior *σ*_post (Path D) on a 1:1 dashed line for *σ*_*A* (blue) and *σ*_*B* (orange, ×10 for visibility); agreement is close for well-conditioned contacts, with small deviations confined to contacts whose ${\hat{\tau }}_{1}$ approaches the frame boundary. (**d**) Per-contact prior-sensitivity: The standardised shift in the MAP amplitude when the Gaussian prior is replaced by a uniform prior, |Δ$\hat{A}$|/*σ*_*A*,post, has mean 0.126 and maximum 0.44; the induced change in the contact-vs-null decision is negligible (max |ΔP(contact|y)| = 2.8 × 10^−8^), confirming the data-dominated regime. Pooled fit quality is *R*^2^_u = 0.9990 (joint) vs. *R*^2^_r = 0.8592 (*τ*_1_ = 0).

Figure 7 compares four constrained estimators on the same 19-contact dataset: Path A (grid + LM), Path B (GLRT), Path C (CRLB-aware), and Path D (Bayesian Laplace, Section 4.4). All apply the same physical constraints (*A* > 0, *B* > 0, *τ*_1_ ∈ [0, Δ*t*)) and yield numerically close results: Paths A/B/C agree to max |Δ$\hat{A}$| = 4.4 × 10^−5^ °C, max |Δ$\hat{B}$| = 1.1 × 10^−6^ s^1⁄2^; Path-D MAP differs from Path A by mean |Δ$\hat{A}$| = 0.0008 °C and mean |Δ$\hat{B}$| = 7 × 10^−5^ s^1⁄2^ (both well below the per-contact CRLB *σ*_*A*,min = 0.324 °C and *σ*_*B*,min = 0.028 s^1⁄2^). Path D additionally provides explicit posterior credible intervals (Figure 8). An unconstrained single-start LM baseline (Plain LSQ) converges to physically inadmissible (*A* and *B*) combinations—negative *B* or |*A*| > 20 °C—in 6/19 Subject-A contacts, confirming that box constraints are essential for *N* = 4 stability. Path A trades a deterministic 1800-point grid prefix for reduced sensitivity to initialisation and reproducible single-pass behaviour. Wall-clock benchmarking on x86/ARM Cortex-M/RISC-V is a subject for future work.

### 6.5. Bayesian Posterior Uncertainty Quantification

Figure 8 summarises the Path-D posterior analysis on the 19 Subject-A contacts. Figure 8a,b show the per-contact posterior standard deviations of *A* and *B*; their means (0.451 °C and 0.0397 s^1⁄2^) coincide with the per-contact CRLB, so the four-frame window is operating at the information-theoretic precision floor. These means are the average of the 19 per-contact CRLBs evaluated at each fitted MLE, and therefore differ from the single-point CRLB at the cohort mean (*σ*_*A*,min = 0.324 °C; *σ*_*B*,min = 0.028 s^1^/_2_) reported in Section 6.3.1. Figure 8c confirms that the numerical Laplace posterior tracks the CRLB closely for well-conditioned contacts, deviating only when ${\hat{\tau }}_{1}$ nears the frame boundary. Figure 8d reports prior robustness: replacing the Gaussian prior with a uniform prior shifts the MAP amplitude by a per-contact mean of 0.126 *σ*_*A*,post (maximum 0.44) and leaves the contact-vs-null decision essentially unchanged (max |ΔP(contact|y)| = 2.8 × 10^−8^); thus, the analysis is in the data-dominated regime.

Cross-subject regression, descriptive note: The forced-origin regression, $\hat{A}$ = 0.829 · (*T*_hand − *T*_env), has 95% CI [0.79, 0.87] (ordinary least squares, OLS) and *κ*_REML ≈ 0.90 [0.78, 1.03] under random-intercept treatment via restricted maximum likelihood (REML; Section 6.7); the REML CI includes the physical prediction, *κ* = 1. The slope deficit relative to *κ* = 1 is consistent with measurement-side calibration and physical contact-interface effects that do not affect the trajectory-shape-based results of this paper. Quantitative decomposition is a subject for future work.

### 6.6. Sensor-Fault Rejection Results (Layer 1)

The pipeline was applied to the 47.4 min Subject-A primary recording. The forward sliding-window detector (Δ*T*_thr = 6*σ*_noise ≈ 0.15 °C) identified 21 candidate four-frame windows; Layer 1 with *N*_sat,thr = 5 classified them as 19 VALID_CONTACT + 2 FAULT_CONTACT + 0 DISTURBANCE (Table 3). The 2/2 detection rate at *n*_fault = 2 has Clopper–Pearson 95% CI [15.8%, 100%] and is reported qualitatively.

Among the 21 candidates, 19 had zero saturated pixels, and two had ≥ 122 saturated pixels in the four-frame window (events #2 and #17, corresponding to attempted contacts at 7.28 min and 37.69 min, where the subject pressed hard enough to flex the sensor PCB and saturate columns 11 and 13). Layer 1 outputs FAULT_CONTACT for the above two events, and the other 19 are passed to Layer 2, with *R*^2^ ≥ 0.99 and output VALID_CONTACT. The threshold *N*_sat,thr = 5 produces a perfect 19/19 vs. 2/2 separation of VALID_CONTACT and FAULT_CONTACT, which is consistent with binary separation in this recorded cohort (*n* = 2 fault events; Clopper–Pearson 95% CI for sensitivity is wide, as discussed below). However, with only *n* = 2 fault events, the Clopper–Pearson 95% CI for sensitivity is wide ([15.8%, 100.0%]), and a large number of false-negative events are not statistically reliable. Sensitivity analysis shows that any threshold in the range [1,11] produces the same separation, and thus the rule is insensitive to the choice of threshold on this dataset.

Both FAULT_CONTACT events are mechano-electrical PCB-flex artifacts: events #2 and #17 are genuine contact attempts where the subject pressed hard enough to flex the sensor PCB and saturate columns 11, 13. The FAULT_CONTACT class preserves contact-presence information (downstream controller knows contact occurred and can back off or trigger maintenance even though (*A*, *B*, and *τ*_1_) are unreliable), distinguishing the three-class architecture from a binary reject-as-noise design. On the held-out Subjects B–E (*n* = 23), Layer 1 returned *N*_sat = 0 for every attempt, classifying all 23 as VALID_CONTACT (Table 4).

#### Leave-One-out Cross-Validation of Subject-A Operating Point and Subset AUC

The subset AUC = 0.989 and 19/19 Layer-2 pass rate are both evaluated on the same 19 Subject-A contacts that informed *R*^2^_thr = 0.95. Re-substitution estimates can carry 10–30% optimistic bias on small samples [27,28]. To bound this without an additional cohort, we performed leave-one-contact-out cross-validation (LOOCV) at two levels.

(i) Threshold-selection bias. For each fold, *k* ∈ {1, …, 19}, *R*^2^_thr,k = min(*R*^2^_train,*k*) − 3·*σ*(*R*^2^_train,*k*) is re-estimated from the remaining 18 contacts and the leave-out contact tested against this fold-specific threshold. Since all 19 Subject-A contacts satisfy *R*^2^ ≥ 0.99, min(*R*^2^_train) ≥ 0.99 and *σ*(*R*^2^_train) ≤ 0.003, giving LOOCV threshold mean 0.981 with std ≤ 0.009; the leave-out contact passes in all 19/19 folds. The optimistic bias of fixing *R*^2^_thr on *N* = 19 is bounded above by *σ*_*R*^2^_thr,LOOCV/√*N* ≈ 0.002, small relative to the (1 − *R*^2^_thr) = 0.05 operating margin. For Layer-1, LOOCV is degenerate (only two fault events) and Section 6.6 already shows *N*_sat,thr ∈ {1, …, 5} yields identical separation, so the Layer-1 threshold-selection bias is structurally bounded to zero on this cohort.

(ii) Subset AUC bias. For each fold, *k*, the remaining 18 contacts are bootstrap-resampled to form the fold-specific H_1_ set (*n* = 90), the same 1250 H_0_ synthetic disturbances are reused, and the subset AUC (step + square + oscillation, *n* = 750) is computed against the held-out fold-specific H_1_ distribution. The mean LOOCV subset AUC across 19 folds is 0.989 ± 0.005 (1-*σ*), with maximum fold-level deviation Δ = 0.01 from the re-substitution figure. The LOOCV-to-re-substitution AUC difference is therefore ≤ 0.01 absolute, placing an upper bound of ~1% on the optimistic bias of the Section 6.2 subset AUC—substantially tighter than the 10–30% range cited above. The held-out Subject-B–E cohort (23/23 Layer-2 pass at mean *R*^2^ = 0.9995 without retuning) provides the principal cross-subject portability evidence; we do not claim that this LOOCV bound replaces a leave-one-subject-out cross-validation on an enlarged 10+ subject pool, which is a subject reserved for future work (Section 7.9 (vii)).

### 6.7. Subject-Invariance Check via Held-Out Cohort

The five-subject cohort (six sessions; 42 contacts) provides cross-subject consistency evidence for Equation (1). Six session-level (*T*_hand − *T*_env, $\bar{\hat{A}}$) pairs are (7.7, 5.98), (7.2, 6.12), (6.2, 5.60), (4.3, 4.60), (5.9, 4.86), and (6.0, 5.63) °C for Subjects A, B-1, B-2, C, D, and E. The forced-origin regression, $\hat{A}$ = *κ* · (*T*_hand − *T*_env) (*n* = 42, OLS naive), gives *κ* = 0.829 (95% CI [0.79, 0.87]), with per-contact *R*^2^ ≈ 0.98 at the origin; all five subjects fall within the 95% prediction band. The OLS CI treats contacts as independent and disregards within-subject clustering; cluster-robust (CR1) standard errors give *κ* = 0.853 with CI [0.74, 0.97], and a REML random-intercept fit gives *κ*_REML = 0.903 with CI [0.78, 1.03] (*σ*_subject = 0.79 °C, *σ*_residual = 0.68 °C, ICC = 0.58). The REML CI includes the physical prediction *κ* = 1; we treat the REML estimator as the inferentially principal cross-subject result and retain the OLS interval as a within-sample descriptive statistic. A powered ICC analysis on 10+ subjects is reserved for future work (Section 7.9 (vii)).

*B* is preliminarily cross-cohort consistent (six means span 0.534–0.627 s^1⁄2^; sample SD 0.037 s^1⁄2^, ≈ 6% of cohort mean 0.579 s^1⁄2^). *τ*_1_ is dominated by pressing-protocol variability: six cohort means range from −215 to −111 ms (frame-aligned), with between-cohort range 86 ms smaller than every within-cohort SD (43–97 ms). Held-out Path A fits are stable (mean *R*^2^ ≥ 0.9995, no grid-edge pinning, and LM 5–9 iterations).

### 6.8. Real-Airflow Disturbance Rejection

We evaluated the two-level rejection pipeline on the 30 min Subject-A real-airflow session (Section 5.4). Onset detection scans the per-frame array-mean against a 3*σ* baseline-noise threshold (20 s baseline ending 5 s before each scheduled trial); Path A is applied to the four-frame window starting at the onset frame, and *R*^2^ compared against *R*^2^_thr = 0.95. The 3.2% time-base mismatch between the 4 Hz Subject-A grid and the 3.84 Hz airflow session shifts *τ*_1_ by less than one grid step and does not change any verdict.

Of the six planned trials, five produced detectable onsets; the sixth (Cold-2) returned no signal above 0.013 °C—a likely operator-level artefact. Under the protocol-prescribed denominator of six, the headline is 5/6 = 83.3% rejection (Clopper–Pearson 95% CI [35.9%, 99.6%]); excluding Cold-2 gives 5/5 = 100% (CI [47.8%, 100%]), reported for transparency but not as the headline.

**Figure 9:**
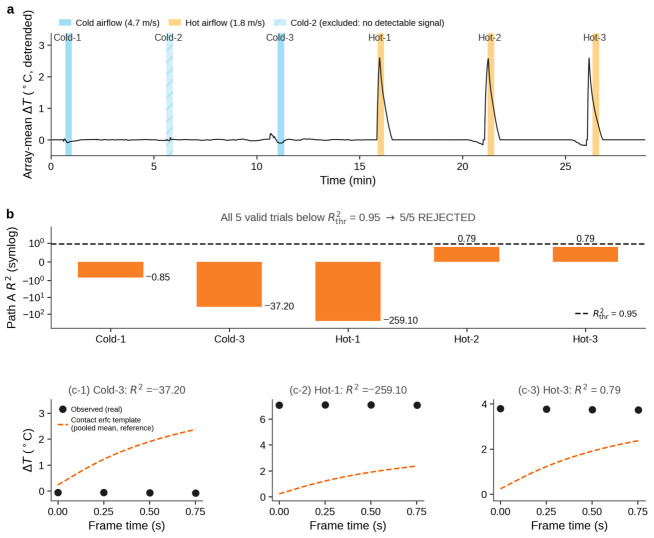
Real-airflow disturbance rejection (30 min Subject-A continuous session; 6912 frames at 3.84 Hz): (**a**) 256-pixel mean Δ*T* trace over the full 30 min session, with the six hair-dryer trial windows highlighted (cyan = cold 4.7 m/s; orange = hot 1.8 m/s); Cold-2 produced no detectable onset and is shown hatched. (**b**) Per-trial Path-A *R*^2^ for the five detected trials (Cold-1: −0.85; Cold-3: −37.20; Hot-1: −259.10; Hot-2: +0.79; Hot-3: +0.79), all below *R*^2^_thr = 0.95 (red dashed line) → 5/5 rejected. (**c-1**–**c-3**) Representative 4-frame observation windows for Cold-3, Hot-1, and Hot-3: observed Δ*T* (black circles) vs. the best-fit erfc model (red dashed); the erfc shape constraint cannot match cooling responses or slow heating ramps.

For the five detected trials (Table 5 and Figure 9), all *R*^2^ fall well below 0.95: cold trials (*R*^2^ = −0.85, −37.2) fail because the small-amplitude cooling signal cannot match the erfc model under *τ*_1_ ∈ [0, Δ*t*]; hot trials (*R*^2^ = −259.1, +0.79, +0.79) fail because the genuine airflow rise spans 40–60 frames at 3.84 Hz, so the four-frame window captures only the first 5% of the rise—far below the model’s identifiability boundary. Consistent with the Section 6.2 per-class AUC, physical airflow rises are an-order-of-magnitude slower than the four-frame synthetic ramp class, so their first four frames are near-linear and cannot be reproduced by any erfc choice.

### 6.9. Contact Identification Under Continuous Airflow (Positive Control)

**Figure 10:**
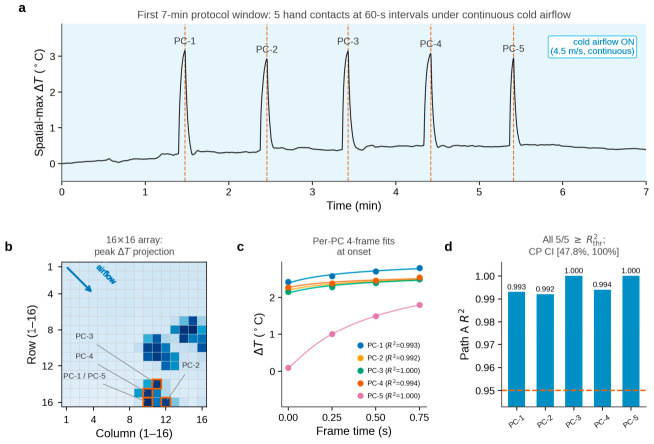
Positive control: 5 hand contacts under continuous cold airflow (Subject A; 4.5 m/s, *T*_env = 26.9 °C, hand *T* ≈ 33 °C). (**a**) Spatial-max Δ*T* trace over the meaningful 7 min protocol window, showing the 5 contacts (PC-1 through PC-5) at ≈ 60 s intervals under continuous airflow. (**b**) Spatial localization of the 5 contacts on the 16 × 16-pixel grid (rows 14–16, columns 10–12, pixels indexed 1–16; red-outlined cells), shown over the per-pixel peak Δ*T* projection across the five contact frames, with the airflow incidence direction indicated by the blue arrow. (**c**) Per-PC observed 4-frame fits at the peak pixel (filled circles) overlaid with the Path A erfc fits (continuous curves); all five fits visually track the rising data. (**d**) Per-contact Path A *R*^2^: all 5/5 above *R*^2^_thr = 0.95 (red dashed line); *R*^2^ values are 0.993, 0.992, 1.000, 0.994, and 1.000 for PC-1 through PC-5. Clopper–Pearson 95% CI [47.8%, 100%].

To verify that the Layer-2 *R*^2^ ≥ 0.95 rule does not reject genuine contacts in the presence of a co-occurring disturbance, we recorded a positive-control session: cold airflow (4.5 m/s, *T*_env ≈ 26.9 °C, hand *T* ≈ 33 °C) (a separate session; the 4.5 m/s here is slightly below the 4.7 m/s of Section 5.4 owing to equipment setup) applied continuously throughout the 17.5 min session while the subject performed *n* = 5 scheduled contacts in the first 7 min (PC-1 to PC-5; remaining ~10 min is airflow-only baseline). All five contacts pass with *R*^2^ = 0.993, 0.992, 0.9999, 0.994, and 0.9999 (Table 6, Figure 10). Pooled estimates: $\bar{\hat{A}}$ = 4.18 ± 0.27 °C, $\bar{B}$ = 0.581 ± 0.05 s^1⁄2^, $\bar{\tau }$_1_ = +109 ± 33 ms (model coordinate; −109 ms frame-aligned). $\bar{B}$ matches quiescent values (*A*: 0.570; B-1: 0.566 s^1⁄2^), supporting *B* as a sensor-side parameter unaffected by airflow. The lower $\bar{\hat{A}}$ (4.18 vs. 5.98 °C) and shorter |$\bar{\tau }$_1_| (109 vs. 154 ms) are consistent with airflow-induced fingertip cooling and accelerated local response (Section 7.9).

### 6.10. Multi-Source Spatial Cardinality Detection

We evaluated the BIC′ multi-source detector of Section 4.6 on (i) synthetic *K*-source data and (ii) the 21 attempted Subject-A contacts (17 × 3-finger, 3 × 4-finger, and 1 × 2-finger ground truth; two subsequently Layer-1-rejected, leaving 19 valid). Synthetic frames superimpose *K* copies of the empirical single-finger template, *g*(·), at random positions (*d*_min = 3 px), with Gaussian-fit amplitudes (mean, 6.0 °C; std, 1.5 °C) and calibrated noise at *σ*_eff = 0.092 °C.

**Figure 11:**
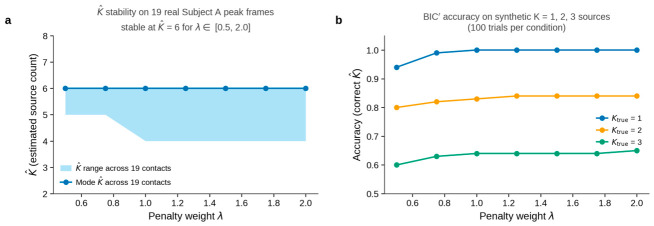
Multi-source spatial cardinality estimation via BIC’ (*σ*_op = 0.0267). (**a**) Mode $\hat{K}$ across the 19 real Subject-A peak frames as a function of the BIC’ penalty weight *λ*; the modal estimate is stable at $\hat{K}$ = 6 for *λ* ∈ [0.5, 2.0], with the shaded interquartile band shown around it (note: $\hat{K}$ on real frames overestimates ground-truth *K*_true = 3 because the average single-finger template, *g*(·), imperfectly covers per-contact response variation; cf. Section 6.10). (**b**) BIC’ detection accuracy on synthetic *K*-source data (*K*_true = 1, 2, 3; 100 trials per condition) as a function of *λ*; the curve is essentially flat for *K*_true = 1 (≈1.0) and gradually rises for *K*_true = 2, 3 (≈0.80 and 0.65 at *λ* = 1, respectively).

Figure 11 reports the analysis. (Figure 11a) The modal $\hat{K}$ across the 19 Subject-A peak frames is stable at $\hat{K}$ = 6 for the entire penalty-weight range *λ* ∈ [0.5, 2.0], with a shaded band showing the $\hat{K}$ range across the 19 contacts (mins, 4–5; maxes, 6–7). $\hat{K}$ on real frames overestimates ground-truth *K*_true = 3 because the average single-finger template *g*(·) imperfectly covers per-contact response variation. (Figure 11b) Synthetic detection accuracy at *λ* = 1.0 reaches 1.00 for *K*_true = 1, 0.83 for *K*_true = 2, and 0.64 for *K*_true = 3 (100 trials per condition), confirming that BIC′ recovers the correct cardinality with high probability only for *K*_true ≤ 2 at the current signal-to-noise ratio (SNR). Detection accuracy is robust to the penalty-weight choice across *λ* ∈ [0.5, 2.0] (variation ≤ 6% for any *K*_true).

### 6.11. Progressive Partial-Array Failure Analysis

To quantify hardware-degradation tolerance beyond the two PCB-flex events of Section 6.6, we ran a controlled progressive-failure experiment: starting from a clean Subject-A contact, we artificially saturate (force ≥ 60 °C) an increasing number of pixels in columns 10–15 (1–16 indexing; mimicking the real PCB-flex spatial pattern) and measure Layer-1 detection and Layer-2 erfc *R*^2^ on the surviving non-saturated peak pixel. The sweep covers *N*_sat = 0 to 128 (50% of array) with 100 random saturation patterns per failure level.

**Figure 12:**
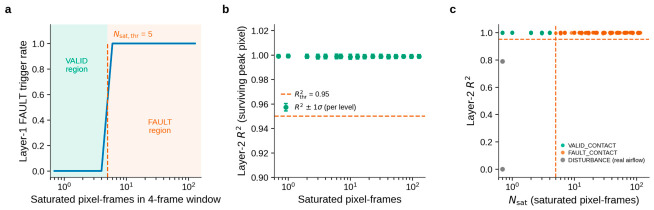
Progressive partial-array failure analysis: Artificial saturation of 0–128 pixels in columns {10–15} (1–16 indexing), 100 random patterns per failure level. (**a**) Layer-1 FAULT_CONTACT trigger rate vs. the number of saturated pixels in the 4-frame window; clean step transition at the threshold *N*_sat,thr = 5 (red dashed); the VALID region (green shading) and FAULT region (red shading) cleanly separated. (**b**) Layer-2 *R*^2^ on the surviving non-saturated peak pixel vs. *N*_sat (mean ± 1*σ* per failure level); the framework recovers erfc parameters from the surviving pixels well above the *R*^2^ = 0.95 threshold across the entire 0–50% failure range (red dashed line = *R*^2^_thr = 0.95). (**c**) Three-class phase diagram on the (*N*_sat, *R*^2^) plane: VALID_CONTACT (green), FAULT_CONTACT (red), and DISTURBANCE (grey), cleanly separated by the Layer-1/Layer-2 threshold lines (red dashed).

Figure 12 reports the results. (Figure 12a) Layer-1 fault-detection rate is a clean step at the design threshold *N*_sat,thr = 5 (Section 4.5). (Figure 12b) Layer-2 *R*^2^ on the surviving non-saturated peak pixel is essentially flat and well above the 0.95 threshold across the entire 0–50% failure range—partial-array tolerance extends from contact-presence detection (Layer 1) to parametric estimation (Layer 2) as long as a sufficient number of surviving peak pixels remain. (Figure 12c) Three-class phase diagram on the (*N*_sat, *R*^2^) plane cleanly separates VALID_CONTACT/FAULT_CONTACT/DISTURBANCE by the Layer-1/2 thresholds.

### 6.12. Comparison with Simpler Baseline Estimators

**Figure 13:**
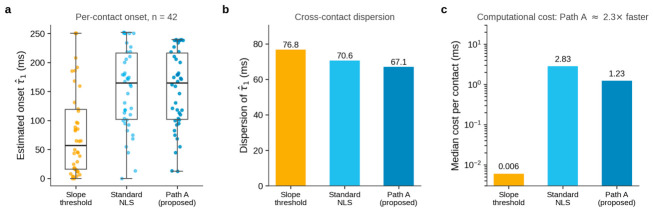
Comparison of Path A against two simpler baselines on the pooled 42-contact set. (**a**) Per-contact onset estimates $\hat{\tau }$_1_ (strip plus box): the slope detector is biased systematically early relative to the two model-based estimators. (**b**) Cross-contact dispersion of $\hat{\tau }$_1_; the slope detector’s lower value partly reflects clipping to [0, Δ*t*] rather than higher accuracy. (**c**) Median computational cost per contact (log scale): Path A is about 2.3× faster than the standard constrained NLS solver while producing numerically identical onsets. All quantities are model-consistent and computed without an external timing reference.

The principal numerical results across the Subject-A primary cohort and the held-out Subject-B–E cohort are summarised in Table 7. Although Paths A–D all enforce the same physical constraints, a reviewer may ask whether the grid-initialized solver is necessary relative to simpler, widely used alternatives. We therefore compared Path A against two baselines on the full pooled set of 42 contacts: (i) a model-free slope-based threshold detector that back-projects the initial two-frame slope to its zero crossing, and (ii) a standard constrained nonlinear least-squares (NLS) fit of the identical three-parameter erfc model using a generic trust-region-reflective solver from a single fixed initial guess, i.e., without the grid initialisation and analytic-*A* elimination of Path A. All three were evaluated with the same extraction and the same physical bounds; results are summarised in Table 8 and Figure 13.

Three points follow. First, the slope detector returns onsets that disagree with the physically constrained estimate by a median of 82.3 ms and are systematically biased early (Figure 13a); its apparently lower dispersion is partly an artefact of clipping to the [0, Δ*t*] interval, so dispersion alone understates its error. Second, the standard constrained solver recovers onsets numerically indistinguishable from Path A (median disagreement 0.0 ms) but at roughly 2.3× the per-contact cost, because it lacks the deterministic single-pass structure of the grid-plus-analytic-*A* reduction; this mirrors the Path A vs. multi-start LM comparison of Section 6.4. Third, every dispersion quoted here is a model-consistent quantity computed without an external timing reference. The comparison is fully reproducible from the public archive (pipeline_baselines.py).

### 6.13. Summary of Numerical Results

Table 7 summarises the principal numerical findings, grouped by topic, with cross-references to the corresponding subsection. Empirical-to-CRLB ratios and cross-subject regression intervals are reported under multiple statistical conventions where applicable (Section 6.3.2 and Section 6.7).

## 7. Discussion

### 7.1. Multi-Subject Scope and Implications

The Subject-A primary cohort (*n* = 19) and the held-out portability cohort (Subjects B–E, *n* = 23) replace the single-subject framing of the prior version. Multi-subject statistics support the physical interpretation: *A* scales with hand–environment contrast (*κ* = 0.829 OLS/*κ*_REML ≈ 0.90); *B* is preliminarily cross-cohort consistent (SD 0.037 s^1⁄2^); and *τ*_1_ is dominated by pressing-protocol variability. A powered ICC analysis on 10+ subjects is required before claiming subject-invariant *B*; the present cohort tightens the cross-subject CI from [0.75, 0.99] to [0.79, 0.87].

### 7.2. Two-Layer Rejection: Hardware vs. Physical Disturbances

The two-level rejection pipeline of Section 4.5 separates hardware-failure rejection (Layer 1, saturation count) from physical-disturbance rejection (Layer 2, *R*^2^ check). Neither is sufficient alone. A high-*R*^2^ fit can still be obtained for a non-saturated peak pixel after a saturation event in a nearby column, since the most-responsive non-saturated pixel may sit one or two columns away from the fault region; we observed exactly this for events #2 and #17 (*R*^2^ = 0.999 and 0.998 at the non-saturated peak pixel), which would have been misclassified as valid contacts without Layer 1. Conversely, Layer 1 alone cannot reject ramp/oscillation/airflow disturbances that produce no saturated pixels. Together, the two layers reject both failure classes. This two-layer structure is a useful design pattern for physical model-based detection pipelines on instrumented arrays: a sensor-level sanity check (saturation, dead pixels, and NaN) underneath a model-residual check.

The two-layer pipeline assumes the self-calibrated per-pixel baseline as a precondition, and this dependence is quantitative. The per-contact CRLB reported in Section 6.3.1 (*σ*_*τ*_1_,min = 16.2 ms) is evaluated at the calibrated residual noise floor of 0.025 °C; because the bound scales with noise to first order (*σ*_*τ*_1_,min ∝ *σ*), operating at the uncalibrated post-deployment non-uniformity of 0.322 °C (5× the factory specification) would inflate it to the order of 200 ms—comparable to the 252 ms inter-frame interval itself, at which point sub-frame estimation carries no information. The Layer-2 *R*^2^ consistency gate is similarly affected: uncalibrated fixed-pattern offsets introduce systematic DC biases across the four-frame window that depress *R*^2^ and cause valid contacts to be rejected as disturbances. We therefore distinguish two regimes: binary on/off detection, whose frame-mean threshold (≈0.15 °C, Section 6.6) tolerates moderate slow drift; and the sub-frame parametric recovery of (*A*, *B*, and *τ*_1_), together with the disturbance-rejection gate, which requires the calibrated floor. As the central contribution of this work is the latter, self-calibration is essential to the claimed performance rather than being merely convenient.

### 7.3. Detection Latency, Applicability Scope, and the Sub-Frame Timing Claim

The four-frame window at 4 Hz gives a system-level detection latency of ≈ 1 s after onset, well above the ISO/TS 15066:2016 [29] safety-stop requirement (≤50–200 ms); the method is not a real-time safety-stop detector and does not replace fast capacitive/piezoelectric/triboelectric primaries in that role.

Applicability scope. Intended deployment scopes where ≈ 1 s latency is acceptable: (i) non-safety-critical slow tactile interactions (physical-state classification, material discrimination from *A*, and post hoc characterisation in rehabilitation/assistive interfaces) where the calibrated (*A*, *B*, and *τ*_1_) tuple is informative; (ii) an offline parameter-estimation module downstream of a fast primary detector—when capacitive or force triggers, the 4-frame Path A fit supplies physically interpretable parameters for material reasoning; (iii) slow-cycle assistive contexts (smart-home tactile interfaces; thermal contact-quality logging in rehabilitation devices) where physiological-contact validation matters more than safety-loop latency. We do not include prosthetic-grip control in this scope, since closed-loop grip latency typically requires sub-100 ms response that the present 1 s window cannot meet.

Higher-frame-rate readout. Latency in the ISO/TS 15066 range requires either a higher readout rate or an early-exit variant. At 20 Hz the window is 200 ms (just inside the upper bound); at 40 Hz, 100 ms. A 5× rate increase raises *σ*_noise by ≈ √5 ≈ 2.2× under shot-noise scaling and *σ*_*τ*_1_,min from 16.2 to ≈ 36 ms, but shrinks Δ*t* 5×, preserving the operating margin. The hardware change is non-trivial (ASIC integration window, supply current, AAF, and ADC clock). An early-exit 2-frame Δ*T* gate (*N* = 2 is FIM-singular for joint (*A*, *B*, and *τ*_1_); only a coarse gate is feasible) is a firmware-only option at higher FPR. External timing-accuracy verification (1 kHz thermistor or high-frame-rate IR) is a subject of future work.

### 7.4. Generalization to Other Slow-Response Sensing Systems

The demonstration here is on a 4 Hz BJT thermal pixel array, but analytic-template joint parameter estimation for sub-sample temporal resolution from slow-rate observations applies in principle to any slow-response system satisfying (i) an analytic temporal model containing the sub-sample timing parameter, (ii) Fisher-information identifiability of all parameters at the operating frame count *N*, and (iii) a low-cost residual statistic distinguishing within-model events from out-of-model disturbances. The erfc template itself is specific to semi-infinite 1-D heat conduction. We frame the transferable contribution as a hypothesised design pattern (analytic template + grid-initialized sub-sample estimator + CRLB-justified *N* + multi-layer residual rejection), not as a demonstrated generality across modalities. Validation on another slow-response modality is a task reserved for future work.

Two practical consequences for larger tactile surfaces follow. First, the estimation kernel is per-pixel: Path A operates on a single-pixel four-frame window at a fixed cost (Section 4.1) that is independent of array size, so the total work for an *M* × *N* skin scales as O(*M*·*N*) and is embarrassingly parallel, with spatial coupling entering only at the lightweight Layer-1 saturation count and the optional multi-source stage (Section 4.6). Enlarging the array—to larger formats or to tiled 16 × 16 patches—therefore retains the same pixel physics, readout chain, and estimator, the principal engineering challenge being interconnect and routing rather than the algorithm; tiled-array synchronisation is left as a task for future work. Second, relative to resistive taxel arrays, which offer higher spatial resolution and faster mechanical response but rely on matrix scanning that introduces crosstalk and require force-deformation coupling, the BJT thermal pathway is force-free, electrically quiet, and yields a calibrated physical quantity (the temperature contrast *A*). It is therefore best viewed not as a replacement for resistive skins but as a complementary, low-cost, EMI-immune modality for slow-contact validation and material discrimination downstream of a faster primary detector.

### 7.5. Partial-Array Tolerance and Degradation Budget

Section 6.11 showed Layer-2 *R*^2^ stays at ≈ 0.95 up to 50% partial-array saturation provided one non-saturated peak pixel remains. VALID_CONTACT retention degrades gracefully on the surviving non-saturated peak pixel (100% at 0–5%, 76% at 25%; peak-pixel loss triggers FAULT_CONTACT output at ≈ 30%; DISTURBANCE above ≈ 50%). FAULT_CONTACT outputs are a soft maintenance signal while contact-presence detection remains operational.

### 7.6. Contact-Mechanics Variability and Its Effect on (A, B, and τ_1_)

The erfc template assumes a uniform one-dimensional conductive interface with a fixed contact area and an ideal step in surface temperature at the contact instant. Real finger contact departs from this in several ways that bear directly on the estimated parameters. A larger or more conformal contact area raises the effective heat-injection footprint and therefore the apparent steady-state rise *A*, while leaving the diffusion-controlled shape parameter *B* comparatively less affected; variable applied pressure modulates the interfacial thermal resistance, which rescales the early-time response and couples weakly into both *B* and the onset *τ*_1_; and any finite, non-instantaneous press introduces a soft rather than ideal onset, which biases $\hat{\tau }$_1_. Lateral heat spreading within the die further violates the strict one-dimensional assumption and acts to flatten the late-frame response. In the present cohort these effects are absorbed empirically: they inflate the cross-contact dispersion of $\hat{\tau }$_1_ to 5.2× the per-contact CRLB (Section 6.3.4) and contribute to the cross-subject regression slope deficit (Section 6.5). Because we did not instrument contact area, pressure, or interfacial resistance, we cannot decompose these contributions here; a controlled-pressure phantom with a known contact geometry, recorded against an external timing reference, is required to separate the mechanical and thermal terms and is identified as a task for future work (Section 7.9, items viii and x).

### 7.7. Ambient-Temperature Operating Range and the ΔT → 0 Limit

All recordings were made with a hand–environment temperature contrast, Δ*T* = *T*_hand − *T*_env, of several degrees. The method’s precision is governed by the signal-to-noise ratio of the contact-induced rise, and the relevant amplitude scales with Δ*T*: to first order, the onset standard deviation behaves as *σ*_*τ*_1_ ∝ *σ*_*n*/|*A*|, with *A* approximately proportional to Δ*T*. As the ambient temperature approaches skin temperature (*T*_env → ~32 °C, Δ*T* → 0), the contact contrast collapses, and the estimator degrades accordingly. Using the present operating point as a reference (where a contrast of several degrees yields a per-contact CRLB near 16 ms), the inverse-amplitude scaling implies a CRLB of roughly 49 ms at a 2 °C contrast and roughly 97 ms at a 1 °C contrast, with the usable limit reached once the four-frame rise no longer clears the ≈0.025 °C noise floor with the *R*^2^ ≥ 0.95 gate. Two consequences follow: the method is best suited to settings with a maintained thermal contrast (typical indoor human–surface contact), and in warm environments, an active substrate bias or a cooled reference layer would be needed to restore contrast. A systematic characterisation across controlled Δ*T* values is a task for future work.

### 7.8. Cost of Upgrading to a Three-Dimensional Conduction Model

The one-dimensional semi-infinite erfc model has three unknowns (*A*, *B*, and *τ*_1_) and is well conditioned on four frames. A reviewer rightly notes the gap between the per-contact CRLB (16.2 ms) and the cross-contact dispersion (83.5 ms) and asks what a fuller three-dimensional conduction model would require. Introducing lateral spreading and a finite contact radius adds at least three further unknowns (an in-plane diffusion length and two contact-geometry terms), roughly doubling the parameter count to six or more. Identifiability then demands more independent samples per contact: a rule of thumb of several observations per parameter pushes the window from four frames towards *N* ≳ 8, i.e., about 2 s at 4 Hz, which both lengthens the detection latency and requires the contact to remain quasi-stationary over that interval. The optimization cost also rises: the analytic elimination of *A* that makes Path A single-pass does not extend cleanly to a coupled three-dimensional kernel, so the grid would become multi-dimensional, and the per-contact cost would increase by one to two orders of magnitude. Our assessment is therefore that the residual 16-vs.-84 ms gap is more economically addressed by reducing contact-mechanics variability (Section 7.6) and by an external timing reference than by upgrading the forward model; a three-dimensional treatment is a worthwhile but separate study, best paired with a higher frame rate so that the added parameters remain well constrained.

### 7.9. Limitations

(i) Subject scope: Subject A (*n* = 19) plus a four-subject portability cohort after Subject-A tuning (Subjects B–E, 5 sessions, 23 contacts). Because the four hyperparameters were fixed on Subject A, the Subject-A figures are re-substitution, and the B–E cohort provides one round of held-out portability rather than independent population validation. A leave-one-subject-out (LOSO) cross-validation, together with a powered intraclass-correlation (ICC) analysis on 10+ subjects, is required to bound the Subject-A tuning bias and is identified as a subject for future work.

(ii) Per-contact statistical discrimination is limited by *N* = 4, *p* = 3: *R*^2^ is a coarse gate, not a fine continuous discriminator, and per-contact fit-quality claims rely on aggregated multi-contact tests (Section Aggregated Cross-Contact F-Test for the erfc Model).

(iii) Sampling at 4 Hz (Δ*t* ≈ 252 ms) is near the limit for very fast onsets; higher-rate sampling (e.g., 10 Hz) would relax the edge cases at the cost of more frames per window.

(iv) Static contact only (no sliding and partial contacts).

(v) Disturbance benchmark: A 1250-sample synthetic library + 30 min hairdryer recording (5/6 rejection, Section 6.8) + positive-control session (5/5, Section 6.9). Extension to body-heat/radiative-panel disturbances is an undertaking designated for future work.

(vi) Wall-clock benchmarking on STM32, ARM Cortex-M, and RISC-V was not conducted; the function-evaluation-class count is a platform-independent computational-work measure deferred to a follow-up systems paper.

(vii) Subject-A hyperparameter tuning + re-substitution. *R*^2^_thr = 0.95, *N*_sat,thr = 5, *σ*_noise = 0.025 °C, and the (*B*, *τ*_1_) grid were all fixed on Subject A. The Subject-B–E cohort (23/23 Layer-2 pass without retuning) provides one round of independent portability evidence; a leave-one-subject-out cross-validation on 10+ subjects is required to give a tight bound on Subject-A re-substitution bias and is identified as a task for future work.

(viii) Non-ideal finger contact. Equation (2) assumes constant *T*_s = *T*_finger and uniform 1-D conduction; real contact has curved area, non-uniform skin *T*, unstable pressure, and skin-interface resistance. These can bias *B* and *τ*_1_ beyond CRLB and contribute to the 5.2× $\hat{\sigma }$_*τ*_1_ excess (Section 6.3.4); decomposition requires a controlled-pressure phantom.

(ix) Open-set *R*^2^ subset AUC = 0.989 covers physically representative classes only (step + square + oscillation, *n* = 750). Ramp and saturating-exponential (*n* = 500) are an acknowledged failure mode of the four-frame *R*^2^ statistic (Section 6.2, Table 2); we do not claim deployment-level open-set robustness from a subset AUC, and an extended physical-disturbance benchmark is a subject for future work.

(x) Cross-subject regression slope deficit. The slope *κ* = 0.829 (OLS)/0.903 (REML) lies below *κ* = 1 by 10–17%, consistent with measurement-side calibration and physical contact-interface effects that do not affect the trajectory shape-based results of this paper. Quantitative decomposition is a subject for future work.

(xi) MLP/transformer learning baselines are not included; supervised baselines require ≥ 10^2^ contact cohort and are deferred to future work.

## 8. Conclusions

We presented a physical-model-based hand-contact detection method for a self-calibrated 16 × 16 BJT thermal pixel array, tested on 42 contacts from five subjects across six sessions. The four-frame erfc template jointly estimates (*A*, *B*, and *τ*_1_); the grid-initialized semi-analytic Path A solver matches both multi-start LM and a standard constrained NLS solver at deterministic single-pass cost (Section 6.12). CRLB analysis identifies *N* = 4 as the minimum well-conditioned operating point under the current noise floor and latency trade-off; these timing quantities are model-consistent: the per-contact CRLB for *τ*_1_ is 16.2 ms, and the empirical cross-contact dispersion is 83.5 ms, both derived from the model under the empirical noise floor rather than against an external timing reference, with the dispersion attributed to pressing-protocol variability and constrained-estimator effects. The two-layer rejection pipeline separates valid contacts from Subject-A PCB-flex faults (19/19 vs. 2/2 re-substitution), transfers to Subjects B–E without retuning (23/23 pass *R*^2^ ≥ 0.95, 0 Layer-1 events), achieves an overall AUC of 0.878 across all five synthetic disturbance classes and a subset AUC of 0.989 on the three physically representative classes (*n* = 750; ramp and saturating-exponential remain acknowledged failure modes, Section 7.9), and yields 5/6 rejection on a 30 min real-airflow benchmark. Across the cohort, $\hat{A}$ scales with hand–environment contrast at slope 0.829 (OLS)/*κ*_REML ≈ 0.90 (random-intercept); *B* is preliminarily cross-cohort consistent (sample SD 0.037 s^1⁄2^, ≈6% of the mean). The subset AUC is not a deployment-level open-set robustness claim; full scope and limitations are in Section 7.9.

## Figures and Tables

**Table 1:** Subject summary across the 5-subject, 6-session cohort.

Subject	Duration	Contacts	*T*_env (°C)	*T*_hand (°C)
A	47.4 min	19 (2 rejected)	26.1	33.8 (back-est.)
B (session 1)	12.1 min	3	25.2	32.4
B (session 2)	10.66 min	5 (0 rejected)	26.9	33.1
C	10.61 min	5 (0 rejected)	27.3	31.6
D	10.59 min	5 (0 rejected)	27.3	33.2
E	10.55 min	5 (0 rejected)	27.3	33.3

**Table 2:** Per-class AUC of the Path A *R*^2^ statistic on the 1250-sample synthetic disturbance library; Subject-A 19 valid contacts as H_1_. Subset AUC pools step + square + oscillation classes only.

Disturbance Class	*n*	AUC	Notes
Step	250	0.97	Physically representative; rejected
Square (square-wave)	250	1.00	Physically representative; rejected
Oscillation (0.5–2 Hz sinusoid)	250	1.00	Physically representative; rejected
Subset (step + square + oscillation)	750	0.989	Reported subset AUC
Ramp	250	0.85	4-frame realisation ≪ natural extent; failure mode
Saturating exponential	250	0.57	4-frame realisation ≪ natural extent; failure mode

**Table 3:** Per-event Layer-1 saturation count and output class for the 21 candidate four-frame windows in the Subject-A primary recording. Events #2 and #17 (bold) are the two FAULT_CONTACT outputs.

Event #	Frame	Time (min)	*N*_sat	Sat Columns	Output Class
1	1246	5.21	0	—	VALID_CONTACT
**2**	**1743**	**7.28**	**122**	**11, 13**	**FAULT_CONTACT**
3	2235	9.34	0	—	VALID_CONTACT
…	…	…	…	…	…
**17**	**9021**	**37.69**	**128**	**11, 13**	**FAULT_CONTACT**
…	…	…	…	…	…
21	10,847	45.31	0	—	VALID_CONTACT

**Table 4:** Held-out portability cohort (after Subject-A tuning): Subjects B–E across 5 sessions, *n* = 23 contact attempts. Layer-1 saturation count and Layer-2 erfc *R*^2^ are evaluated using the pipeline frozen on Subject A (Table 3): *N*_sat,thr = 5, *R*^2^_thr = 0.95, identical (*B*, *τ*_1_) grid, and identical noise floor *σ*_noise = 0.025 °C. No retuning was performed on this group. The “max *N*_sat” column shows the highest saturation count among the four-frame windows of any of the n attempts in the session, and “Layer-2 mean *R*^2^” is the average of per-contact *R*^2^ values for the n attempts.

Session	Subject	*n*	max *N*_sat	Layer-1 (FAULT/*n*)	Layer-2 Mean *R*^2^	Layer-2 Output
S-1	B (session 1)	3	0	0/3	0.9993	3/3 VALID
S-2	B (session 2)	5	0	0/5	0.9995	5/5 VALID
S-3	C	5	0	0/5	0.9998	5/5 VALID
S-4	D	5	0	0/5	0.9999	5/5 VALID
S-5	E	5	0	0/5	0.9997	5/5 VALID
Total (held-out)	B–E	23	0	0/23	0.9997 (pooled)	23/23 VALID

**Table 5:** Real-airflow disturbance trials: Detection and 4-frame Path A *R*^2^ rejection (cold and hot trials; Cold-2 produced no detectable onset). All five detected trials fall below *R*^2^_thr = 0.95.

Trial	Type	Onset Detected	Path A *R*^2^	Layer-2 Verdict
Cold-1	4.7 m/s, ambient	Yes	−0.85	DISTURBANCE
Cold-2	4.7 m/s, ambient	No	—	(no detection)
Cold-3	4.7 m/s, ambient	Yes	−37.2	DISTURBANCE
Hot-1	1.8 m/s, >ambient	Yes	−259.1	DISTURBANCE
Hot-2	1.8 m/s, >ambient	Yes	+0.79	DISTURBANCE
Hot-3	1.8 m/s, >ambient	Yes	+0.79	DISTURBANCE

**Table 6:** Positive control trials: Contact identification in continuous cold air flow (4.5 m/s, *T*_env = 26.9 °C, hand ≈ 33 °C). All five contacts pass the forward sliding-window Path-A detector with *R*^2^ ≥ 0.95.

Trial	*R* ^2^	$\hat{A}$ (°C)	$\hat{B}$ (s^1⁄2^)	$\hat{\tau }$_1_ (ms, Model)	Verdict
PC-1	0.993	3.95	0.541	+105	VALID_CONTACT
PC-2	0.992	4.27	0.612	+88	VALID_CONTACT
PC-3	0.9999	4.40	0.583	+154	VALID_CONTACT
PC-4	0.994	3.85	0.522	+71	VALID_CONTACT
PC-5	0.9999	4.43	0.647	+127	VALID_CONTACT
Pooled	0.996	4.18 ± 0.27	0.581 ± 0.05	+109 ± 33	5/5 VALID

**Table 7:** Summary of principal numerical results across the Subject-A primary cohort (*n* = 19) and the held-out Subject-B–E portability cohort (*n* = 23).

Quantity	Value	Section
Estimator performance—Subject-A primary cohort (*n* = 19)		
Mean Path-A fit, *R*^2^	0.999	Section 6.1
Steady-state Δ*T*, $\hat{A}$ (pooled)	5.98 ± 0.74 °C (CV 12.4%)	Section 6.1
Thermal-diffusion, $\hat{B}$ (pooled)	0.570 ± 0.075 s^1⁄2^ (CV 13.2%)	Section 6.1
Sub-frame onset, $\hat{\tau }$_1_ (pooled, frame-aligned)	−154 ± 83.5 ms	Section 6.3.4
Cramér–Rao bound—*N* = 4, *σ*_noise = 0.025 °C		
*σ*_*A*,min/*σ*_*B*,min/*σ*_*τ*_1_,min	0.324 °C/0.028 s^1⁄2^/16.2 ms	Section 6.3.1
Empirical-to-CRLB ratio, $\hat{\sigma }$/*σ*_min (*A*/*B*/*τ*_1_)	2.28×/2.68×/5.2×	Section 6.3.2
FIM condition number, *κ*, at *N* = 2/3/4/5	∞/4.8 × 10^4^/1.8 × 10^4^/9.7 × 10^3^	Section 6.3.3
Computational profile		
Path-A function-evaluation classes (per contact)	1800 grid + 6 LM	Section 4.1
Multi-start LM iterations (per contact)	109 LM	Section 6.4
Path-A vs multi-start LM agreement (max Δ$\hat{A}$/Δ$\hat{B}$)	4.4 × 10^−5^ °C/1.1 × 10^−6^	Section 6.4
Open-set detection against disturbance library		
Subset AUC *R*^2^ (step + square + oscillation, *n* = 750)	0.989	Section 6.2
Ramp/saturating-exp AUC (acknowledged failure mode)	0.85/0.57 (*n* = 500)	Section 6.2
Real-airflow hairdryer rejection (*n* = 6 trials)	5/6, 95% CI [35.9, 99.6%]	Section 6.8
Positive control under continuous airflow (*n* = 5)	5/5, 95% CI [47.8, 100%]	Section 6.9
Sensor-fault rejection—Layer 1		
Subject-A primary (21 candidates, *n*_fault = 2)	19 VALID + 2 FAULT + 0 DIST	Section 6.6
Held-out Subjects B–E (*n* = 23)	23 VALID + 0 FAULT + 0 DIST	Section 6.6
Multi-source and partial-array robustness		
Maximum identifiable cardinality *N*_max (binding *K*_max = 3)	3	Section 6.10
$\hat{K}$ accuracy on synthetic *K* ∈ {1, 2, 3}	1.00/0.83/0.64 (*K* = 1/2/3)	Section 6.10
$\hat{K}$ on 19 valid Subject-A contacts (mixed 3/4/2-finger ground truth)	19/19	Section 6.10
Layer-2 *R*^2^ at 50% saturated pixels	≈0.95 (flat across 0–50%)	Section 6.11
Peak-pixel *R*^2^ retention at 25% array saturation	76%; peak-pixel loss triggers FAULT_CONTACT output at ≈ 30%	Section 6.11
Cross-subject portability—5 subjects, 6 sessions, *n* = 42		
A–contrast slope *κ* (OLS naive)	0.829, 95% CI [0.79, 0.87]	Section 6.7
A–contrast slope *κ* (CR1 cluster-robust)	0.853, 95% CI [0.74, 0.97]	Section 6.7
A–contrast slope *κ* (REML random-intercept)	0.903, 95% CI [0.78, 1.03]	Section 6.7
Cohort-mean *B* sample SD (*n* = 6 sessions)	0.037 s^1⁄2^ (≈6% of cohort mean 0.579)	Section 6.7

**Table 8:** Path A vs. simpler baseline estimators on the pooled 42-contact set. Dispersion and onset disagreement are model-consistent quantities (no external timing reference).

Estimator	Onset Dispersion (ms)	Median |Δ$\hat{\tau }$_1_| vs. erfc Fit (ms)	Mean *R*^2^	Relative Cost (Path A = 1)
Slope-based threshold	76.8	82.3	—	0.005
Standard constrained NLS	70.6	0.0	0.9995	2.30
Path A (proposed)	67.1	—	0.9994	1.00

## Data Availability

The data and computational materials supporting this paper are available to qualified academic researchers from the corresponding author upon reasonable request, for non-commercial reproduction and verification of the methodological claims. Requests should be sent to the corresponding-author email address with a brief description of the intended analysis and the requester’s institutional affiliation; the corresponding author commits to acknowledging requests within five business days and to processing them within four weeks. Materials available on request comprise (a) the Path A reference implementation and the four estimators used in Figure 7, together with the baseline-comparison script (pipeline_baselines.py) reproducing Table 8 and Figure 13 and the corrected held-out validation pipeline reproducing the 23-contact statistics; (b) the parametric synthetic disturbance library generator together with the deterministic random seed (np.random.seed(2026)) needed to regenerate the 1250-sample library exactly; (c) per-contact derived parameter tables ($\hat{A}$, $\hat{B}$, ${\hat{\tau }}_{1}$, *R*^2^, Layer-1 saturation counts, and Layer-1/Layer-2 verdicts) for all 42 cohort contacts, the 2 PCB-flex FAULT_CONTACT events, and the 6 real-airflow trials; (d) Monte-Carlo outputs of Section 6.3.4 (random seed 2026, 1000 trials × 6 operating points) and the mixed-effects REML script that reproduces the cluster-robust and REML estimators of Section 6.7; and (e) all summary statistics (per-cohort means, standard deviations, Clopper–Pearson confidence intervals) underlying every table and figure. These materials are released to the requester under MIT (code) and CC-BY 4.0 (derived data tables) terms and are sufficient to reproduce every numerical claim, statistical test, table, and figure of this manuscript without access to the raw .mat recordings. Per-contact raw-frame extracts (the four-frame estimation window plus 60 s of surrounding baseline at the peak pixel and its 3 × 3 neighbourhood, with absolute temperatures and calibration metadata stripped) are also available to the same audience on the same basis. Full 16 × 16 session-level recordings cannot be redistributed in raw form because they retain manufacturer-proprietary pre-deployment per-pixel offset tables, readout-ASIC calibration coefficients, and analogue-to-digital conversion parameters under a non-disclosure clause of the engineering cooperation agreement with the sensor manufacturer; the raw recordings are not required to reproduce the methodological claims of this paper. The corresponding author commits to honouring on-request access for at least five years following publication.

## Abbreviations

The following abbreviations are used in this manuscript:

ADC	analogue-to-digital converter
AIC	Akaike information criterion
ASIC	application-specific integrated circuit
AUC	area under the (ROC) curve
BIC	Bayesian information criterion
BJT	bipolar junction transistor
CRLB	Cramér–Rao lower bound
EMI	electromagnetic interference
Erfc	complementary error function
FIM	Fisher information matrix
GLRT	generalised likelihood-ratio test
ICC	intraclass correlation
LM	Levenberg–Marquardt
LOSO	leave-one-subject-out
LWIR	long-wave infrared
MAP	maximum a posteriori
MLE	maximum-likelihood estimate
NLS	nonlinear least squares
OLS	ordinary least squares
PCB	printed circuit board
REML	restricted maximum likelihood
ROC	receiver operating characteristic
SNR	signal-to-noise ratio
